# Tailoring Low-Cost Granular Activated Carbons Intended for CO_2_ Adsorption

**DOI:** 10.3389/fchem.2020.581133

**Published:** 2020-11-19

**Authors:** Marcos Juliano Prauchner, Silvia da Cunha Oliveira, Francisco Rodríguez-Reinoso

**Affiliations:** ^1^Instituto de Química, Universidade de Brasília, Campus Darcy Ribeiro, Brasília, Brazil; ^2^Departamento de Química Inorgánica, Universidad de Alicante, Alicante, Spain

**Keywords:** capture of CO_2_, CO_2_ separation, activated carbon, adsorption, chemical activation, physical activation, flue gas, biogas

## Abstract

Physical adsorption on activated carbons has shown to be a very attractive methodology for CO_2_ separation from flue gas streams and biogas. In this context, the goal of this work was to prepare granular activated carbons intended for CO_2_ adsorption from an abundant and low-cost biomass residue (coconut shell) by using practical and cost-effective procedures. By the first time, parameters involved in chemical activation with dehydrating agents (H_3_PO_4_ or ZnCl_2_) and/or physical activation with CO_2_ were systematically screened in depth in order to obtain materials with improved performance for CO_2_ adsorption on a volume basis. Compared with the commonly used mass basis, the data expressed on a volume basis are very important for industrial applications because they permit to estimate the efficiency of a fixed bed adsorption column. The work permitted to prepare granular activated carbons with a blend of relatively high gravimetric CO_2_ uptake and bulk density, so that high volumetric CO_2_ uptakes were attained. The highest values were 2.67 and 1.17 mmol/cm^3^ for CO_2_ pressures of 1.0 and 0.15 bar, respectively. It is remarkable that the obtained results were similar to those reported by other authors for carbons chemically activated with KOH, the activation methodology that has been widely claimed as the one that produce ACs with the best performances for CO_2_ adsorption, but which involves severe restrictions. Therefore, the present work can be considered a very important step in paving the way toward making CO_2_ adsorption an each time more interesting technology to reduce the emissions of anthropogenic greenhouse gases.

## Introduction

Global warming has been considered as one of the most serious problems of the twenty-first century because it has threatened global economies and the environment. In spite of existing controversies, the problem has been widely attributed to the increase in the atmospheric concentration of greenhouse gases (GHGs), of which CO_2_ and CH_4_ are the most important ones. Anthropogenic CO_2_ is extensively emitted due to the burning of fossil fuels in power plants and other combustion engines (McMillan et al., 2005). In turn, the main source of atmospheric CH_4_ is the biogas generated by anaerobic digestion of biodegradable organic matter by microorganisms in sewage treatment plants, landfills and digestion plants to treat manure and industrial/agricultural wastes. In this sense, the capture of CO_2_ at large fixed emission sources and the recovery and utilization of biogas have been considered crucial strategies for reducing the concentration of GHGs in the atmosphere, as depicted in the next two Subsections.

### CO_2_ Capture

In spite of the efforts to replace fossil fuels by renewable fuels, the former certainly will continue to be the primary global energy source at least in the foreseeable future. Therefore, the capture of CO_2_ at large fixed emission sources such as thermoelectric power plants and industrial plants (e.g., steel mills, refineries and cement kilns) has been increasingly employed as a strategy for reducing the emissions of the gas. After separated from the flue gas stream (which contains mainly CO_2_ and N_2_, with typical CO_2_ concentrations between 10 and 15%), the CO_2_ is compressed into a supercritical fluid, transported and safely deposited in the ground or an ocean-bedrock sediment layer (the so-called carbon capture and storage approach—CCS) (Huaman and Jun, 2014; Leung et al., 2014; Lee and Park, 2015; Rashidi and Yusup, 2016; Leeson et al., 2017; Bui et al., 2018). Alternatively, efforts have been directed toward CO_2_ recycling and reuse in industry, agriculture, oil recovery, and energy production (the so-called carbon capture and utilization approach—CCU) (Kikuchi, 2003; Aresta and Dibenedetto, 2007; Leung et al., 2014; Bui et al., 2018; Fu et al., 2019).

### Biogas Upgrading and Use

Biogas consists primarily of CH_4_ (40–75%) and CO_2_ (15–60%), besides minor quantities of other components such as H_2_O, H_2_S, siloxanes, hydrocarbons, NH_3_, O_2_, CO, and N_2_. Due to its high CH_4_ content, biogas is a potential fuel for the generation of electrical power and heat. In this sense, after an appropriate pre-treatment, the biogas can be used as a natural gas substitute in applications such as car fuel, production of hydrogen by steam reforming for fuel cells, and syngas production. Besides removing contaminants harmful to the natural gas grid, appliances and end-users, the referred pre-treatment (the so-called biogas upgrading or enrichment) aims to reduce the CO_2_ content in the fuel in order to increase its calorific heat accordingly to the envisaged application. After that, the final product typically contains 95–97% of CH_4_ and 1–3% of CO_2_, being referred to as biomethane or bio-natural gas (Alonso-Vicario et al., 2010; Ryckebosch et al., 2011; Yang et al., 2014).

At this point, it is important to mention that CH_4_ has a global warming potential around 28 times higher than CO_2_ tacking a 100 years base (Myhre et al., 2013). Therefore, the conversion of CH_4_ into CO_2_ by biogas combustion is by itself very positive for the mitigation of the greenhouse effect. Furthermore, if one takes into account that the burned biogas replaced a fossil fuel, thus this positive effect becomes even higher.

### CO_2_ Separation

The separation of CO_2_ is a critical step in both biogas upgrading and CCS/CCU systems, and it is still an economic burden. In the separation of CO_2_ from flue gas in CCS/CCU systems, chemical absorption by amine/ammonia solutions (amine/ammonia scrubbing) is nowadays the most employed technology due mainly to the high process efficiency (up to 98%). However, the technique presents some serious shortcomings, which are most related to the high-energy consumption for solvent regeneration and pumping, corrosion of equipment and toxic emissions (Zhao et al., 2012; Rashidi and Yusup, 2016). Concerning biogas upgrading, physical absorption by water under pressure (the so-called pressurized water scrubbing, PWS) is the most used technique for CO_2_/CH_4_ separation. PWS is a simple and not expansive technique that presents high efficiency and low CH_4_ loss, besides permitting the simultaneous removal of H_2_S. However, clogging due to bacterial growth, the huge water consumption and low flexibility toward variation of input gas are serious existing drawbacks (Ryckebosch et al., 2011; Nie et al., 2013; Niesner et al., 2013; Xu et al., 2015).

Taking into account the above-mentioned shortcomings related to amine/ammonia scrubbing and PWS, the development of alternative cost-effective technologies to separate CO_2_ from flue gas streams and biogas is highly desirable. Such alternatives include cryogenic separation, membrane separation, biological removal, and adsorption (Presser et al., 2011; Leung et al., 2014; Lee and Park, 2015). Amongst them, physical adsorption on solid porous sorbents has been considered the most appealing option because of the low energy required and ease of applicability over a relatively wide range of temperatures and pressures (Chaffee et al., 2007; Shafeeyan et al., 2010; Songolzadeh et al., 2012; Chen et al., 2013; Azmi and Aziz, 2019).

### CO_2_ Adsorption on Activated Carbons

Numerous materials have been investigated for CO_2_ adsorption, which includes zeolites, silica, metal-organic frameworks (MOFs), alkali-based and metal oxide-based adsorbents, porous polymers and carbonaceous materials (Songolzadeh et al., 2012; Lee and Park, 2015; Rashidi and Yusup, 2016). Amongst them, activated carbons (ACs) are a promising option (Silvestre-Albero et al., 2011, 2014; Sevilla et al., 2019) because they: present stability in terms of resistance to thermal, mechanical and chemical strength; are safe for the environment; can be easily produced from abundant and cheap raw materials such as coal, biomass and petroleum residues, which is of overwhelming importance in large-scale applications; are hydrophobic, which is important to avoid competitive adsorption of water; can have the pore morphology easily designed by an appropriate choice of activation conditions; have fast adsorption/desorption kinetic; establish weak interactions with CO_2_, which makes easy and less energy demanding the discharge process by thermal or pressure modulation (Wang et al., 2013; Parshetti et al., 2015; Li and Xiao, 2019; Singh et al., 2019).

According to the literature, the overall majority of ACs intended for CO_2_ adsorption have been prepared by chemical activation with KOH. The reason is that this procedure is renowned for rendering carbons with remarkably high specific surface area and microporosity, so that the resulting adsorbents have usually shown the highest gravimetric CO_2_ uptakes, as it can be verified from the data in the Supplementary Table 1. Notwithstanding, we have at least three considerations to make about this issue, as depicted in the sequence.

(i) Firstly, quite large amounts of KOH (a caustic and highly corrosive chemical) are employed in the activation process. Usual KOH:precursor ratio are in the range of 1 to 4, which can be considered unviable for a large-scale production (see, just as few examples, the works of Wahby et al., 2010; Deng et al., 2014; Parshetti et al., 2015; Haffner-Staton et al., 2016; Hong et al., 2016; Huang et al., 2019; Kutorglo et al., 2019; Li et al., 2019).

(ii) Secondly, activation with KOH invariably renders carbons in the powdered form. Therefore, before being used in fixed bed adsorption columns, the material should be extruded or conformed into monoliths. Besides implying additional costs, these procedures usually require the employment of a binder, which obstructs part of the porosity (Jordá-Beneyto et al., 2008; Munusamy et al., 2015).

(iii) Thirdly, the overwhelming majority of authors have only considered the CO_2_ uptake on a gravimetric basis. However, considering that the adsorbent has to be confined in a given column with limited volume, so it is much more relevant, from an application point of view, to evaluate the data on a volume basis rather than on a mass basis. This issue was firstly considered by Silvestre-Albero et al. (2011); however, as far as we know, since then only Li et al. (2019), Haffner-Staton et al. (2016), and Marco-Lozar et al. (2012) have reported the CO_2_ uptake of ACs on a volumetric basis.

### Purpose and Scope of This Paper

Within the above-depicted scenario, the goal of the present work was to prepare biomass-based granular ACs with the highest volumetric CO_2_ uptakes as possible (at atmospheric and subatmospheric pressures), but employing activation procedures that are practical and cost-effective for a large-scale production. In this sense, we used an abundant and cheap feedstock: coconut shell, a residual biomass. Furthermore, the adsorbents were prepared in the granular form because it is most practical and inexpensive. One could argue that the interparticle space of granular adsorbents reduces the volumetric adsorption uptake. Of course, it is true; however, it is needed to have in mind that, on the other hand, the existence of some free space is essential to permit adequate gas diffusion throughout a packed column.

As activating methodologies, we investigated in depth the chemical activation with dehydrating agents (H_3_PO_4_ or ZnCl_2_) and the physical activation with CO_2_. Although there are some scarce works reporting the use of these approaches in the production of ACs intended for CO_2_ adsorption (Presser et al., 2011; Vargas et al., 2011; Bae and Su, 2013; Ello et al., 2013; Balsamo et al., 2014; Ludwinowicz and Jaroniec, 2015; Munusamy et al., 2015; Ahmed et al., 2019) and the reported gravimetric CO_2_ uptakes are, in a general way, lower than those verified for KOH activated carbons (compare the data in Supplementary Table 1), none of the existing works concerned a systematic study in which the activation conditions are screened in order to achieve materials with improved performances. Further, the results were always considered only on a mass basis instead of a volume basis.

Finally, it is worthy to highlight that, along the present work, efforts were also directed toward the comprehension of the relationships between the procedures employed to prepare the ACs, the resulting pore morphology and the performance for CO_2_ adsorption of the obtained materials.

## Materials and Methods

### Activation Procedures

A sample of dried endocarp of coconut shell (Cocos nucifera) was used as raw material. After crushing and sieving, the fraction in the range of 2.00–2.83 mm was used in all preparations. Some characteristics of the precursor were previously published (Prauchner and Rodríguez-Reinoso, 2012). The carbonization and physical activation procedures were carried out in a horizontal tubular furnace. For carbonization, the samples were heated (2.0°C/min) under N_2_ flow (100 mL/min) up to the desired temperature (2 h).

For chemical activation, the precursor was first impregnated with an aqueous solution of the chemical (2.0 mL per gram of precursor). The solution concentration was adjusted to provide the desired mass ratio of phosphorous or zinc to the precursor (these ratios will be termed as *X*_*P*_ or X_Zn_, respectively). The impregnated material was then carbonized up to 450 or 500°C (for activation with H_3_PO_4_ or ZnCl_2_, respectively) and washed to remove the chemical. Some additional details on the chemical activation procedures were previously reported (Prauchner and Rodríguez-Reinoso, 2012).

Concerning the complementary carbonization, it is worthy to highlight that the chemically activated samples were firstly prepared as described above and, in a subsequent and independent step, carbonized again, this time up to 850°C.

For physical activation, the starting material (which had always been previously carbonized up to 850°C) was heated (5°C/min) under N_2_ flow (100 mL/min). When reached the activation temperature (750°C), the gas flow was switched to CO_2_ (100 mL/min) and the temperature was kept for the period of time necessary to achieve the desired burn-off (weight loss due to the gasification with CO_2_).

### Activated Carbon Labels

The ACs were labeled according to their preparing procedure and the following rules:

a) A carbonization step was indicated by the letter C followed by the maximum reached temperature.

b) For chemical activation, the letter P or Z was used to indicate the chemical (H_3_PO_4_ or ZnCl_2_, respectively), followed by the X_P_ or X_Zn_ value multiplied by 100.

c) For physical activation, the letter B was used followed by the respective burn-off (in percentage).

Thus, for example, the Z15.C850.B20 sample concerns the material that was chemically activated with ZnCl_2_ with a X_Zn_ of 0.15, followed by carbonization up to 850°C and subsequent physical activation with CO_2_ up to a burn-off of 20%.

In turn, in order to represent an entire series of samples, the letter “X” was used in the place of the parameter that varied within that series (temperature, chemical loading or burn-off). In this way, for example, the P09.C850.BXX series comprises all the activated carbons produced by chemical activation with H_3_PO_4_ with a X_P_ 0.09, followed by carbonization up to 850°C and subsequent gasification with CO_2_ up to different burn-offs.

### Activated Carbon Characterization

The pore morphology was evaluated from the excess adsorption isotherms of N_2_ (−196°C) and CO_2_ (0°C) recorded up to 1.0 bar in a volumetric automatic system Omnisorb 610. Before any experiment, samples were degassed (10^−4^ Pa) at 350°C for 4 h.

The N_2_ adsorption isotherms were used to determine the specific surface area (*S*_*BET*_) and the micropore volume (*V*_*mic*_*)* by applying the BET and DR equations, respectively. The volume of liquid N_2_ adsorbed at *p/p*_0_ 0.95 was termed *V*_0.95_ and it was considered to be the sum of *V*_*mic*_ and the volume of mesopores (*V*_*mes*_). Therefore, *V*_*mes*_ was calculated by subtracting *V*_*mic*_ from *V*_0.95_. In turn, the CO_2_ isotherms were employed in the calculation of the ultramicropore volume (*V*_*ult*_) (sometimes also termed narrow micropore volume). Finally, the software Autosorb 1 was used to generate pore size distribution curves from the N_2_ and CO_2_ adsorption isotherms (PSD-N_2_ and PSD-CO_2_, respectively) by using the non-linear density functional theory (NLDFT).

The gravimetric CO_2_ uptakes were determined from the CO_2_ adsorption isotherms acquired as above described. In turn, the volumetric CO_2_ uptakes were determined by multiplying the gravimetric uptakes by the sample bulk density (ρ_*b*_). The latter was measured by gently tapping a weighted amount of grains in a graduate cylinder (tap density).

The CO_2_ uptakes were taken at 1.0 and 0.15 bar. These pressures were chosen because they mimic typical conditions for CO_2_ capture from biogas or flue gas streams. For example, if the concentration of CO_2_ in the gas mixture is 15% (a typical value), thus 1.0 bar corresponds to the partial pressure of CO_2_ in a pressure swing adsorption unit operating at 6.7 bar. In turn, 0.15 bar corresponds to the partial pressure of CO_2_ in a vacuum swing or temperature swing adsorption unit operating at atmospheric pressure.

An automatic pycnometer Accupyc 1330 from Micromeritics was used to measure the helium density (ρ_*h*_), which was considered equivalent to the carbon skeleton density. As previously detailed (Prauchner et al., 2016), the gravimetric waste volume (*V*_*w,g*_) was calculated from the following equation:

$$\begin{array}{l} V_{\text{w},\text{g}}=1/\rho_{\text{b}}-V_{0.95}-1/\rho_{\text{h}} \end{array}$$

Thus, the volumetric waste volume (*V*_*w,v*_), determined by multiplying *V*_*w,g*_ by ρ_*b*_, comprises, in a bed filled by the adsorbent, the fraction of volume that corresponds to interparticle spaces plus large pores that are not filled by N_2_ at p/p_0_ = 0.95 and −196°C.

## Results and Discussion

For reasons of space, only some selected isotherms essential for the ongoing discussions are showed in the main text. Nevertheless, the N_2_ adsorption/desorption and CO_2_ adsorption isotherms of all the prepared samples are available as Supplementary Figures 4–8. The data of pore morphology and density are furnished in Table 1. A detailed discussion about porosity development during physical activation with CO_2_ and chemical activation with H_3_PO_4_ or ZnCl_2_ was reported elsewhere (Prauchner and Rodríguez-Reinoso, 2012).

**Table 1:** Data of pore morphology and density.

**Sample**	***S_***BET***_***	***V_***ult***_***	***V_***mic***_***	***V_***mes***_***	***V_**0.95**_***	**ρ*_***He***_***	**ρ*_***b***_***
	**(m^**2**^/g)**	**(cm^**3**^/g)**	**(cm^**3**^/g)**	**(cm^**3**^/g)**	**(cm^**3**^/g)**	**(g/cm^**3**^)**	**(g/cm^**3**^)**
C450	^*^	0.14	^*^	^*^	^*^	1.42	0.575
C850	^*^	0.26	^*^	^*^	^*^	1.89	0.611
C850.B25	939	0.41	0.43	0.03	0.46	2.01	0.500
C850.B35	1,046	0.45	0.49	0.05	0.54	2.04	0.473
C850.B52	1,340	0.46	0.59	0.09	0.68	2.08	0.416
C850.B79	1,987	0.49	0.78	0.23	1.01	2.14	0.323
C850.B94	2,276	0.48	0.81	0.41	1.22	2.19	0.270
P09	869	0.34	0.37	0.03	0.40	1.59	0.536
P15	1,218	0.35	0.46	0.14	0.60	1.66	0.532
P21	1,294	0.29	0.50	0.12	0.62	1.67	0.501
P27	1,620	0.33	0.59	0.21	0.80	1.71	0.458
P30	1,826	0.30	0.62	0.35	0.97	1.73	0.422
P36	2,017	0.37	0.68	0.41	1.09	1.76	0.347
P54	2,212	0.35	0.73	0.66	1.39	1.79	0.281
Z15	719	0.32	0.32	0.01	0.33	1.58	0.544
Z25	1,293	0.37	0.52	0.07	0.59	1.62	0.519
Z32	1,392	0.36	0.55	0.08	0.63	1.65	0.507
Z40	1,700	0.36	0.66	0.14	0.80	1.65	0.445
Z50	1,960	0.38	0.71	0.27	0.98	1.69	0.405
Z65	1,978	0.33	0.73	0.37	1.10	1.69	0.330
P09.C850	665	0.26	0.27	0.03	0.30	1.97	0.680
P09.C850.B22	1,143	0.41	0.48	0.03	0.51	2.04	0.554
P09.C850.B34	1,470	0.43	0.59	0.07	0.66	2.12	0.491
P09.C850.B44	1,945	0.54	0.69	0.11	0.80	2.15	0.445
P09.C850.B54	2,106	0.50	0.76	0.22	0.98	2.17	0.393
Z15.C850	679	0.23	0.31	0.00	0.31	1.95	0.660
Z15.C850.B20	1,096	0.40	0.46	0.00	0.46	2.05	0.583
Z15.C850.B29	1,211	0.48	0.54	0.03	0.57	2.11	0.524
Z15.C850.B36	1,489	0.53	0.65	0.03	0.68	2.14	0.485
Z15.C850.B44	1,599	0.54	0.70	0.07	0.77	2.15	0.438

* *Not determined because the quite narrow porosity of the C450 and C850 samples hindered, at −196°C, the access of the N_2_ molecules at a period of time plausible for the acquirement of the isotherms*.

### Relationships Between Pore Morphology and CO_2_ Uptake

Before presenting and discussing the results verified for each series of carbons, the data of all the prepared samples were analyzed as a whole in order to search for relationships between the pore morphology and the CO_2_ uptake. These relationships will be valuable for subsequent discussions.

In this sense, the gravimetric CO_2_ uptake of all prepared samples were plotted vs. the data of pore morphology. The plots showed no evident relationship between the CO_2_ uptake and the parameters determined from the N_2_ adsorption isotherms (*S*_*BET*_, *V*_0.95_, and *V*_*mic*_; Supplementary Figure 1). On the other hand, it was possible to identify trends of increasing CO_2_ uptake with increasing volume of pores determined from the CO_2_ adsorption isotherms (*V*_*ult*_; Supplementary Figure 2). Notwithstanding the low correlation coefficients (*R*^2^), especially in the case of adsorption at 0.15 bar, these results disclosed that, as already suggested by several authors (Presser et al., 2011; Wahby et al., 2012; Wei et al., 2012; Casco et al., 2013; Sevilla et al., 2013; Silvestre-Albero et al., 2014; Haffner-Staton et al., 2016; Serafin et al., 2017; Jang et al., 2019; Sreńscek-Nazzal and Kiełbasa, 2019), CO_2_ adsorption at low pressure occurs mostly in narrow micropores, which can be more accurately probed by CO_2_ adsorption experiments. Namely, CO_2_ isotherms are usually acquired at much higher temperature (0°C) than N_2_ isotherms (−196°C), which drastically reduce the diffusional restrictions. In addition, the CO_2_ saturation pressure (p_0_) at 0°C is ~35 bar, so that 1.0 bar corresponds to a relative pressure (p/p_0_) of only ~0.03. Therefore, it is usually accepted that, at 0°C and atmospheric pressure, CO_2_ molecules are significantly adsorbed only in narrow micropores, where the proximity of neighboring walls causes an overlapping of adsorption potentials (Rodríguez-Reinoso and Linares-Solano, 1989). This is the reason why the volume of pores determined by applying the DR equation to CO_2_ isotherms at 0°C is considered to correspond to the volume of ultramicropores (d < 0.7 nm, where d is the pore dimension) (Garrido et al., 1987; Rodríguez-Reinoso et al., 1989; Cazorla-Amorós et al., 1996).

**Figure 1:**
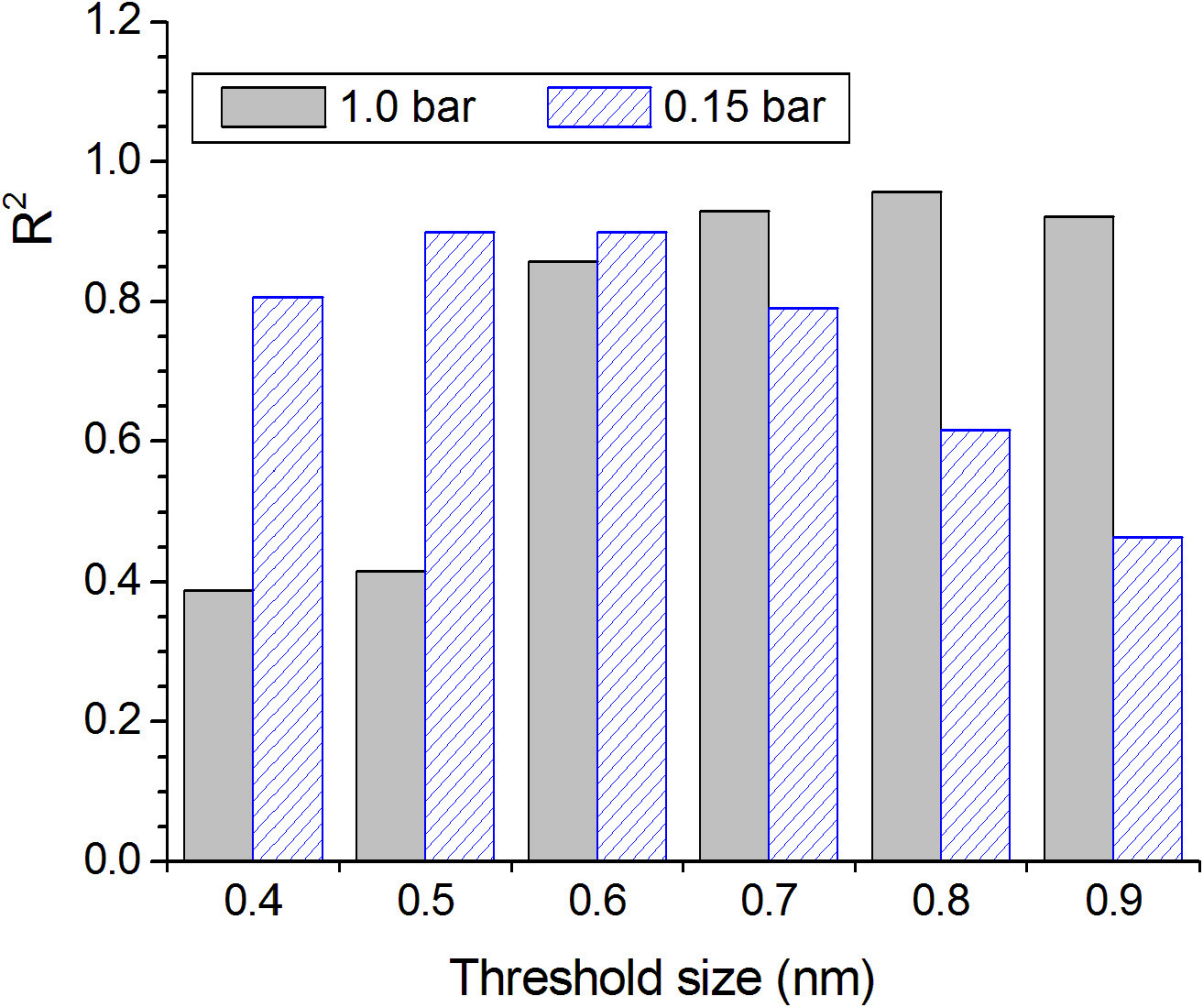
*R*^2^ values verified for the linear fit of the plots of gravimetric CO_2_ uptake at 1.0 and 0.15 bar vs. the volume of pores smaller than a given threshold size.

The above-mentioned results triggered more in-depth studies concerning the CO_2_ adsorption isotherms. In this sense, the gravimetric CO_2_ uptake was plotted against the volume of pores smaller than a certain threshold size determined from the PSDs-CO_2_ curves: 0.4, 0.5, 0.6, 0.7, 0.8, and 0.9 nm (V_<0.4_, V_<0.5_, V_<0.6_, V_<0.7_, V_<0.8_, and V_<0.9_, respectively). The graphs (Supplementary Figure 3) showed that, for a CO_2_ pressure of 1.0 bar, the CO_2_ uptake correlates better with the volume of pores smaller than 0.8 nm, with a *R*^2^ of 0.9573 (Figure 1). In turn, for a CO_2_ pressure of 0.15 bar, the higher *R*^2^ was verified when the data were plotted against *V*_<0.6_, with a *R*^2^ value of 0.8997. These findings permitted to infer that, at 0°C, pores with dimensions in the lower range of ultramicropores are efficiently filled by CO_2_ at pressures as low as 0.15 bar, whereas pores with dimensions in the higher range of ultramicropores need pressures of around 1.0 bar to be effectively filled.

### CO_2_ Uptake

Before going ahead with the discussions, it is worthy to emphasize that the volumetric uptake is determined by multiplying the gravimetric uptake by the adsorbent bulk density and, in a general way, the bulk density decreases with increasing porosity. Therefore, to reach an optimized volumetric uptake, the porosity should be increased in such a controlled way that the density of the adsorbed phase is not significantly reduced. On the otherwise, an eventual increase of the gravimetric CO_2_ uptake can be surpassed by the reduction of bulk density, therefore resulting in a lower volumetric CO_2_ uptake.

#### Carbonization in Absence of Chemicals

**Figure 2:**
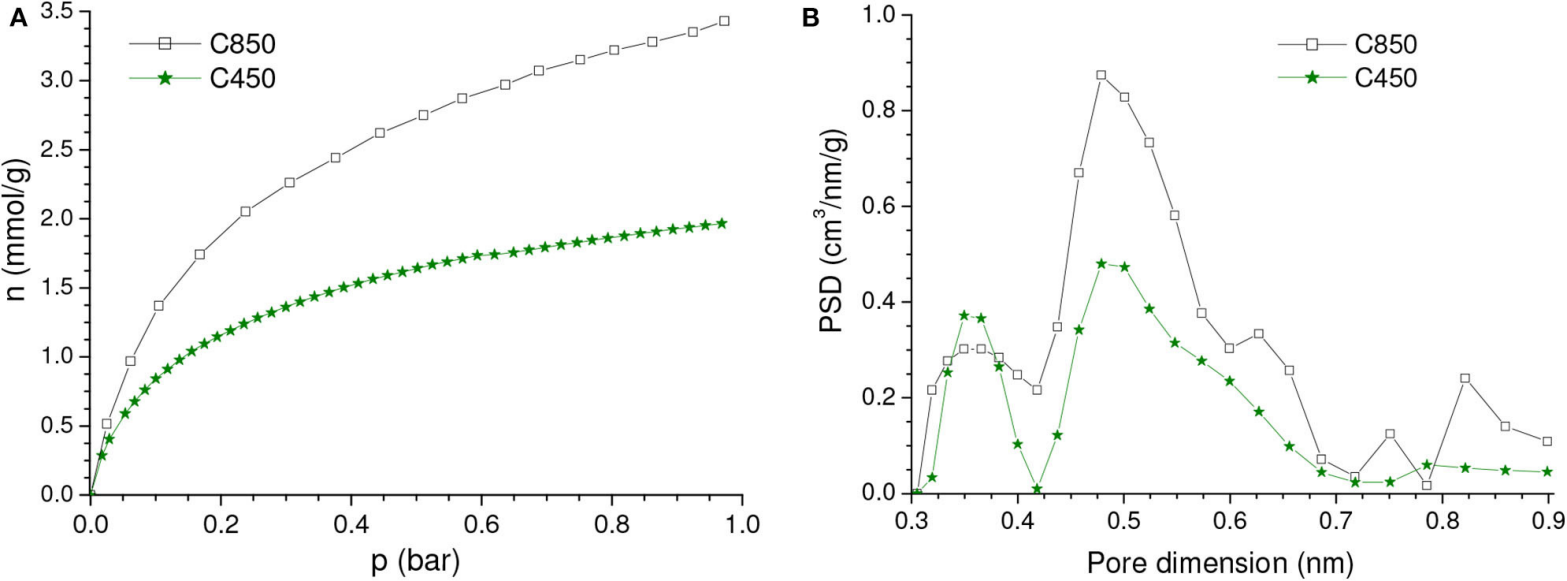
**(A)** CO_2_ adsorption isotherms and **(B)** corresponding PSDs-CO_2_ curves of the samples carbonized up to 450 and 850°C in absence of chemicals.

During the carbonization of lignocellulosic feedstocks in absence of chemicals, volatile matter, aliphatic carbons and heteroatoms are released, while residual carbon atoms are grouped into stacks of flat aromatic sheets cross-linked in a random manner. Thus, the irregular arrangement of carbon sheets gives rise to narrow free interstices that correspond to an incipient porosity (Cândido et al., 2020). Indeed, the sample obtained by coconut shell carbonization up to 450°C (C450 sample) presented a *V*_*ult*_ of 0.14 cm^3^/g (Table 1). The PSD-CO_2_ curve (Figure 2B) showed two main peaks corresponding to pore dimensions in the range of ~0.30–0.42 nm and ~0.42–0.72 nm, with maxima at 0.36 and 0.49 nm, respectively. This narrow porosity resulted in gravimetric CO_2_ uptakes of 1.98 and 1.03 mmol/g at 1.0 and 0.15 bar, respectively. Taking the sample bulk density (0.575 g/cm^3^) into account, the respective volumetric uptakes were calculated as 1.14 and 0.59 mmol/cm^3^.

It is well-known that, during carbonization, pronounced condensation of aromatic rings takes place between around 600 and 800°C, thus resulting in a porosity increase (Prauchner et al., 2005). Indeed, *V*_*ult*_ enhanced from 0.14 to 0.26 cm^3^/g when the carbonization temperature increased from 450 to 850°C (Table 1). Consequently, if compared with the C450 sample, the CO_2_ adsorption isotherm of the C850 sample presented higher CO_2_ uptakes across the entire range of pressures (Figure 2A). At 1.0 and 0.15 bar, the gravimetric CO_2_ uptake increased from 1.98 to 3.47 mmol/g and from 1.03 to 1.64 mmol/g, respectively. Taking the bulk densities into account (which was 0.611 g/cm^3^ for the C850 sample), it was determined that the respective volumetric uptakes increased from 1.14 and 0.59 mmol/cm^3^ to 2.12 and 1.00 mmol/cm^3^.

Concerning the PSD-CO_2_ curve (Figure 2B), C850 also presented two peaks at nearly the same positions as verified for C450, being that the area of the peak with the maximum at ~0.49 nm was pronouncedly higher for the C850 sample. Once the position of the peak did not change, it is possible to conclude that the porosity increase verified when the carbonization temperature increased from 450 to 850°C was due to the creation of new pores rather than by the growth of the existing ones.

#### Physical Activation

During physical activation with CO_2_, the activating gas diffuses throughout the incipient porosity formed during the precursor carbonization and gasifies the walls accordingly to the following reaction: CO_2_ + C → 2CO (Marsh and Rodríguez-Reinoso, 2006). Indeed, Table 1 shows that the porosity gradually increased with increasing burn-off and, consequently, the bulk density decreased.

**Figure 3:**
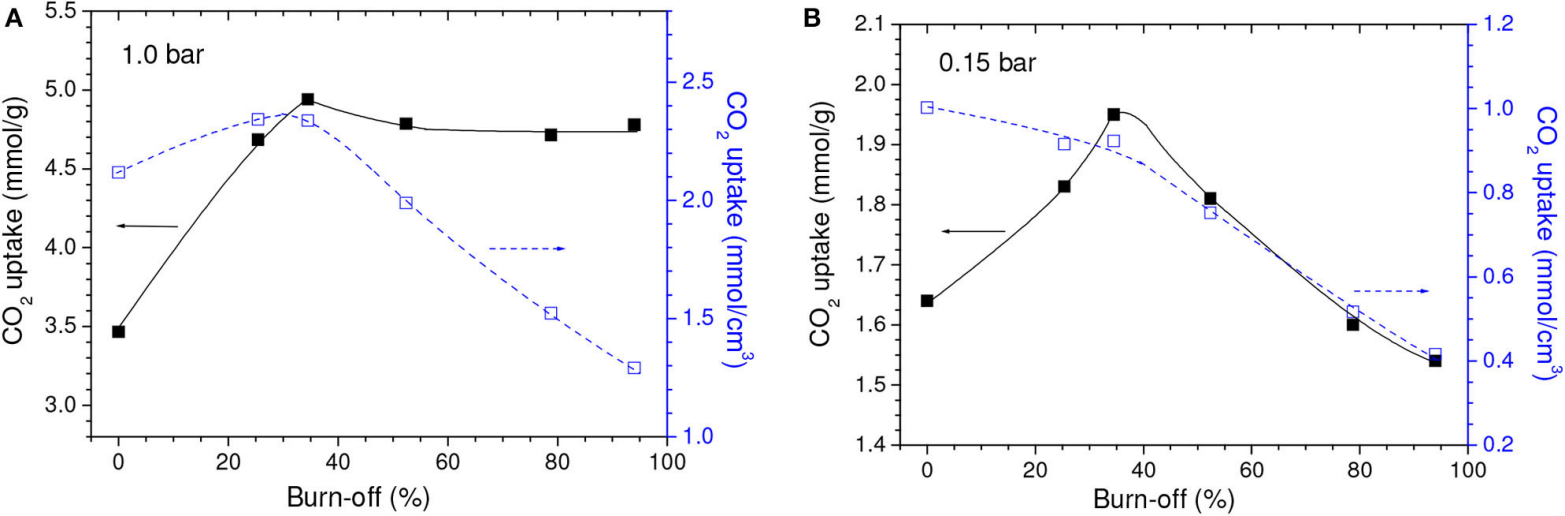
Gravimetric and volumetric CO_2_ uptakes at **(A)** 1.0 and **(B)** 0.15 bar as a function of the burn-off during physical activation with CO_2_.

Even though the volume of micropores plus mesopores (*V*_0.95_) continuously increased, the volume of ultramicropores (*V*_*ult*_) first increased but few changed after a burn-off of around 35% (Table 1). Therefore, taking into account that ultramicropores are more effective for CO_2_ adsorption at atmospheric pressure, the gravimetric CO_2_ uptake at 1.0 bar reached a maximum for the C850.B35 sample, 5.0 mmol/g, and then nearly stabilized (Figure 3A).

**Figure 4:**
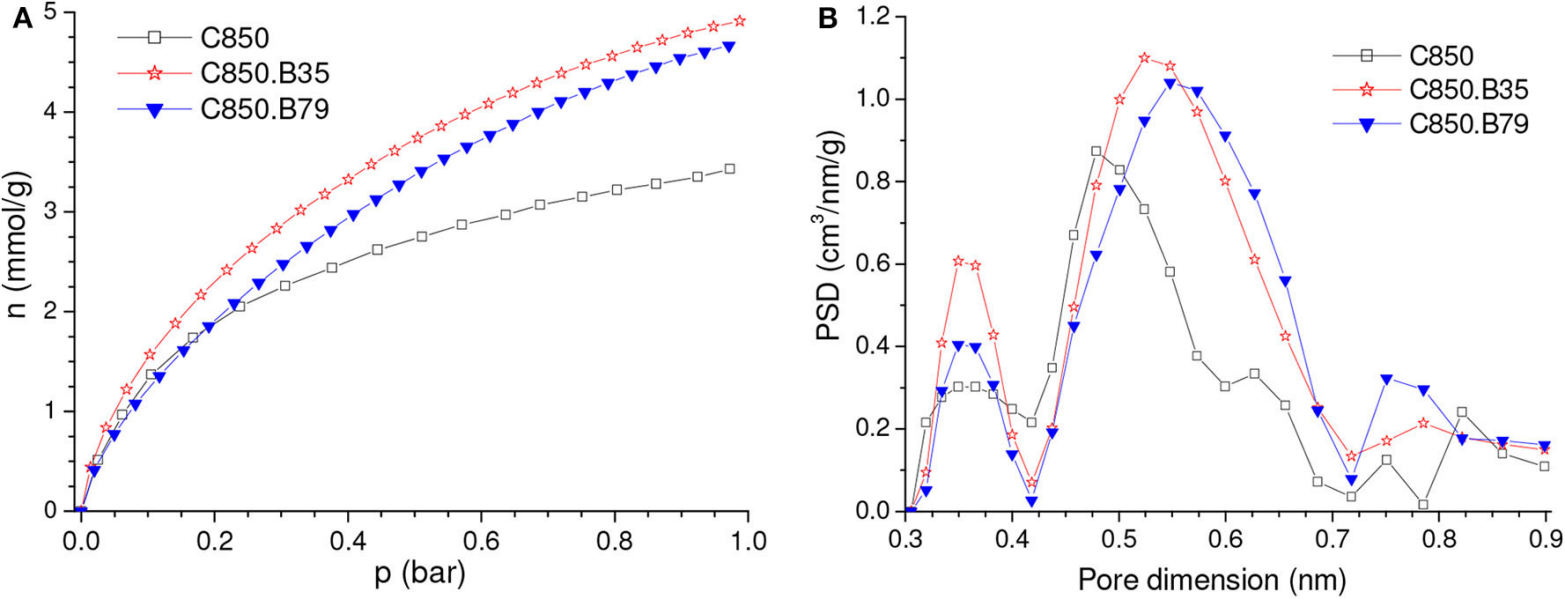
**(A)** CO_2_ adsorption isotherms and **(B)** corresponding PSDs-CO_2_ curves of the carbon obtained by carbonization up to 850°C and some selected samples physically activated with CO_2_.

The isotherms of CO_2_ adsorption and corresponding PSD curves of the C850 and C850.B35 samples (Figures 4A,B) disclose that, up to moderate burn-offs, the increase of porosity took place in both the lower and the higher range of ultramicropores. However, when the gasification was more intense, the volume of pores located in the lower range of ultramicropores diminished, as portrayed by the PSD-CO_2_ curve of the C850.B79 sample. This supposedly occurred because the dimensions of these small pores enhanced along the gasification process, so that they partially moved toward the higher range of ultramicropores. Thanks to this behavior and the fact that CO_2_ adsorption at low subatmospheric pressures is more efficient at small ultramicropores, the gravimetric CO_2_ uptake at 0.15 bar reached a maximum for the B35 sample, ~1.95 mmol/g, and decreased thereafter (Figure 3B).

Concerning the volumetric uptake, different behaviors were observed at 1.0 and 0.15 bar. As Table 1 discloses, the porosity development verified up to a burn-off of around 35% was basically due to increases in the volume of ultramicropores, so that almost all of the gain in porosity was effective for increasing the CO_2_ adsorption at 1.0 bar. Therefore, within this burn-off range, the increase of gravimetric CO_2_ uptake at 1.0 bar surpassed the reduction of bulk density and, consequently, the volumetric CO_2_ uptake reached a maximum (~2.40 mmol/cm^3^) for burn-offs between 25 and 35% (Figure 3A). On the other hand, only the pores located in the lower range of ultramicropores are effective for adsorbing CO_2_ at 0.15 bar. Therefore, even for low activation degrees, the increase in the gravimetric CO_2_ uptake at this pressure was not sufficient to compensate the reduction of bulk density. Consequently, all the physically activated samples had volumetric CO_2_ uptake at 0.15 bar lower than that of the only carbonized sample C850 (which was 1.00 mmol/cm^3^) (Figure 3B). Furthermore, the uptake steadily decreased with increasing burn-off.

#### Chemical Activation

During the carbonization of lignocellulosic precursors impregnated with H_3_PO_4_ or ZnCl_2_, the carbon matrix develops around the chemicals, which leaves the structure in an expanded state. Thus, the subsequent removal of the chemical by leaching makes a pore structure available (Prauchner and Rodríguez-Reinoso, 2012). Indeed, Table 1 shows that chemical activation with H_3_PO_4_ or ZnCl_2_ rendered carbons with higher porosity and lower bulk density than the sample carbonized at similar temperature in absence of chemicals (C450 sample). Further, the higher the chemical loading, the higher the *V*_0.95_ and the lower the bulk density.

**Figure 5:**
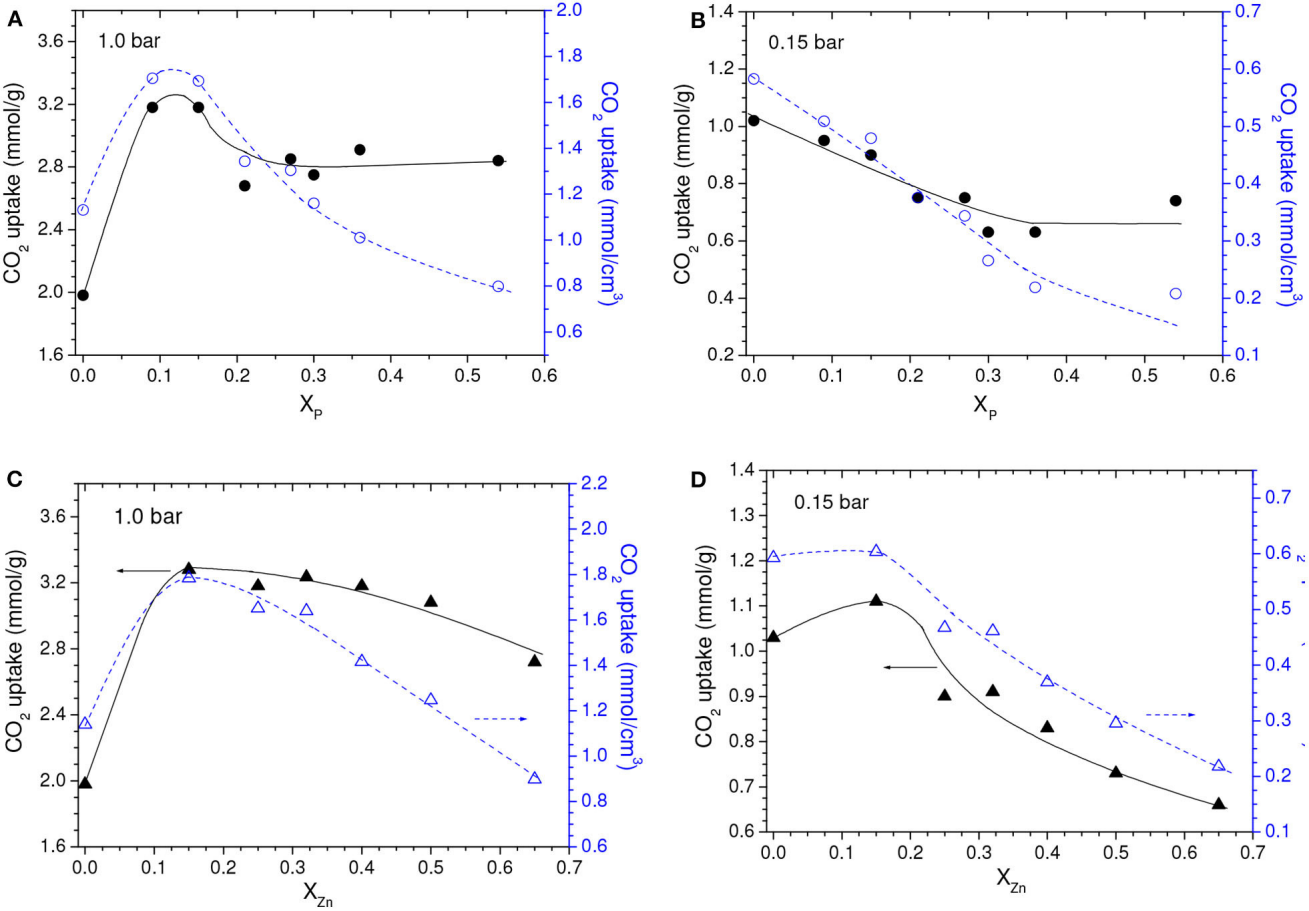
Gravimetric and volumetric CO_2_ uptakes at **(A,C)** 1.0 bar and **(B,D)** 0.15 bar as a function of the chemical loading during chemical activation with **(A,B)** H_3_PO_4_ or **(C,D)** ZnCl_2_. Obs. The points relative to X_P_ and X_Zn_ values of zero correspond to the C450 sample.

In comparison with the C450 sample, the higher porosity of the samples chemically activated with the lowest loadings (P09 and Z15 samples) was primarily due to their higher volume of ultramicropores (compare the data in Table 1). In turn, for higher loadings, *V*_*ult*_ oscillated without a defined trend, while the volume of larger pores increased. Therefore, since CO_2_ adsorption at atmospheric pressure takes place mostly at ultramicropores, the gravimetric CO_2_ uptake at 1.0 bar was abruptly higher for the P09 and Z15 samples (3.2 and 3.3 mmol/g, respectively) if compared with the C450 sample (1.98 mmol/g), and slightly decreased for higher chemical loadings (Figures 5A,C).

**Figure 6:**
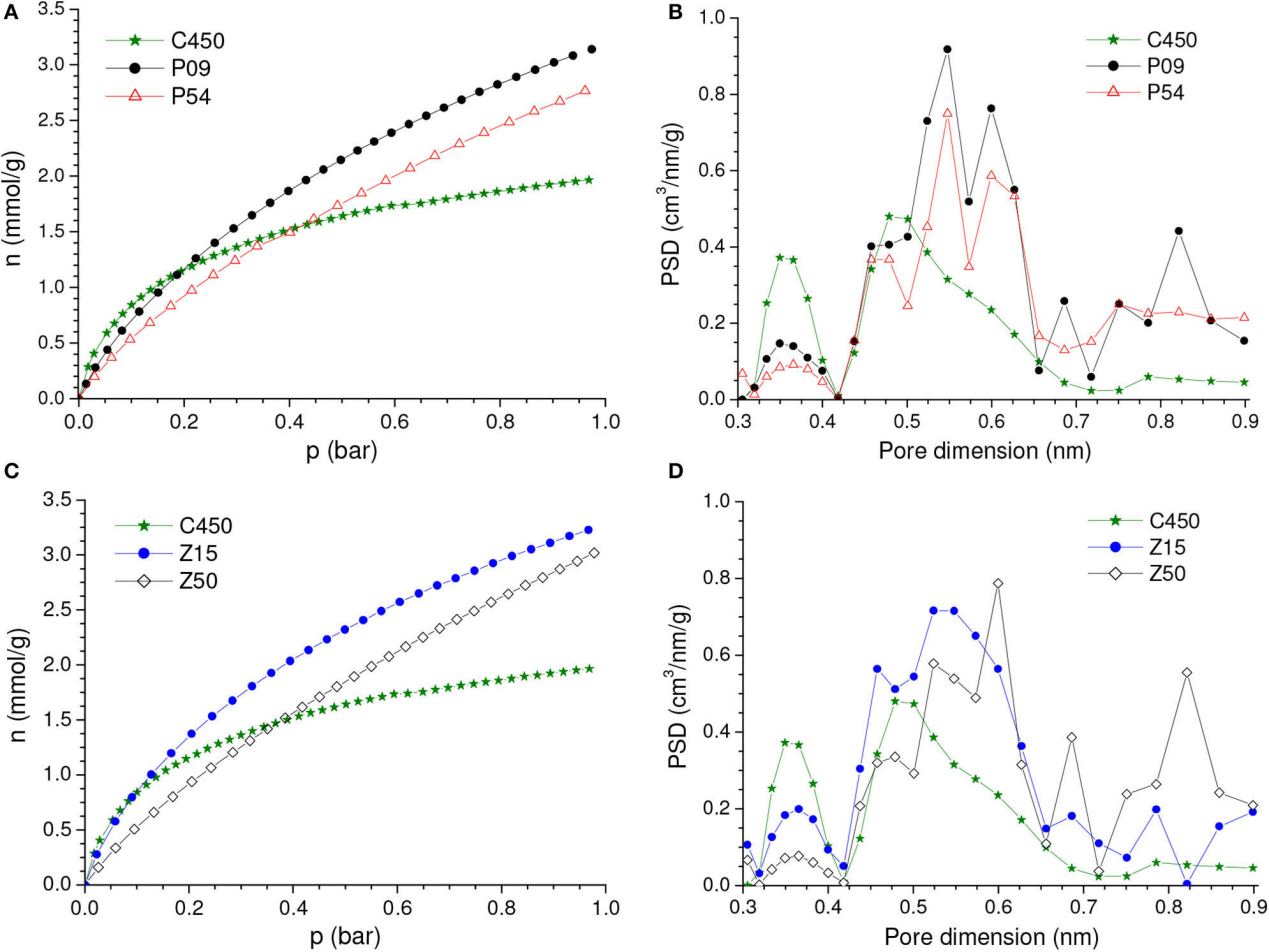
**(A,C)** CO_2_ adsorption isotherms and corresponding **(B,D)** PSDs-CO_2_ curves of the sample obtained by carbonization of coconut shell up to 450°C in absence of chemicals and some selected samples of carbons chemically activated with H_3_PO_4_ or ZnCl_2_.

Conversely to what was observed for a CO_2_ pressure of 1.0 bar, the chemical activation with low chemical loadings did not promote considerable increases in the gravimetric CO_2_ uptake at 0.15 bar (Figures 5B,D). Instead of that, the uptake for the Z15 sample was only marginally higher than the value verified for the C450 sample, while the uptake of the P09 sample was even lower. In addition, the uptake pronouncedly decreased with increasing chemical loading. This profile of behavior can be explained based on the isotherms of CO_2_ adsorption of the chemically activated samples (Figures 6A,C) and respective PSD curves (Figures 6B,D). The curves show that, if compared with carbonization in absence of chemicals, chemical activation provoked a reduction in the volume of pores below ~0.42 nm; the higher the chemical loading, the more intense was this reduction. Thus, since pores located in the lower range of utramicropores are responsible for most of the CO_2_ adsorption at low subatmospheric pressures, the reduction in the volume of pores below 0.42 nm diminished the CO_2_ uptake at 0.15 bar.

The above-mentioned reduction in the volume of pores below ~0.42 nm can be explained as it follows. As already discussed in the Subsection “Carbonization in absence of chemicals”, the irregular arrangement of carbon sheets during the carbonization of non-impregnated biomass generates free interstices that correspond to pores in the lower range of ultramicropores. In turn, if the carbonization is performed in presence of dehydrating agents such as H_3_PO_4_ or ZnCl_2_, the chemical decomposes the lignocellulosic chains and the resulting fragments reorganize and give rise to a more ordered carbon structure. Therefore, at the same time that the chemical acts as a physical template for the development of larger pores, the material reorganization supposedly reduces the occurrence of free interstices that correspond to small ultramicropores.

#### Comparing the Activations With CO_2_, H_3_PO_4_, and ZnCl_2_

**Figure 7:**
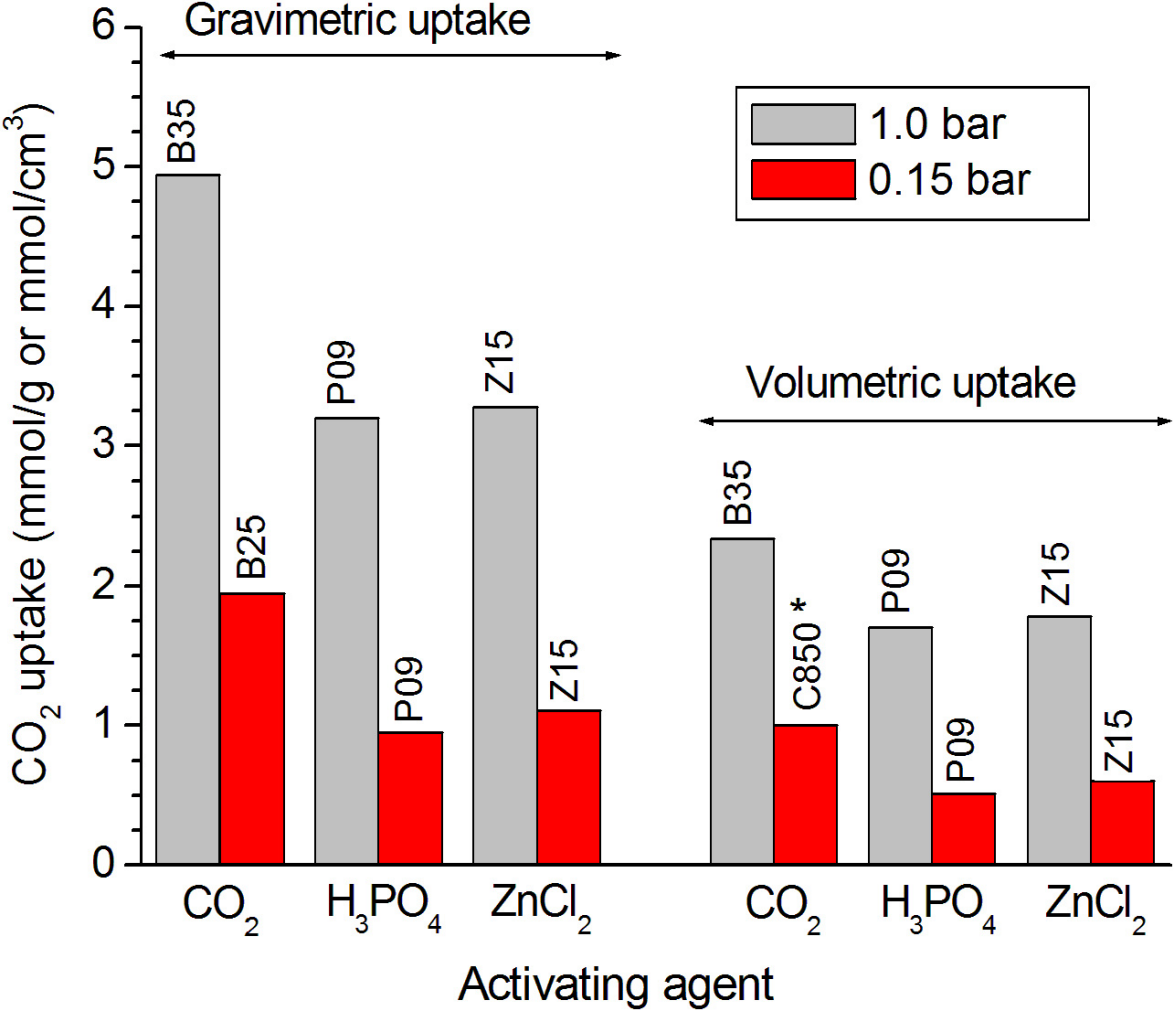
Plotting of the highest CO_2_ uptakes verified for each series of carbons prepared by physical activation with CO_2_ and chemical activation with H_3_PO_4_ or ZnCl_2_. *For the series of carbons physically activated with CO_2_, the lower the burn-off, the higher the volumetric uptake at 0.15 bar; thus, the maximum value for this series was considered here as being the value verified for the only carbonized sample C850, which would correspond to a burn-off zero.

**Figure 8:**
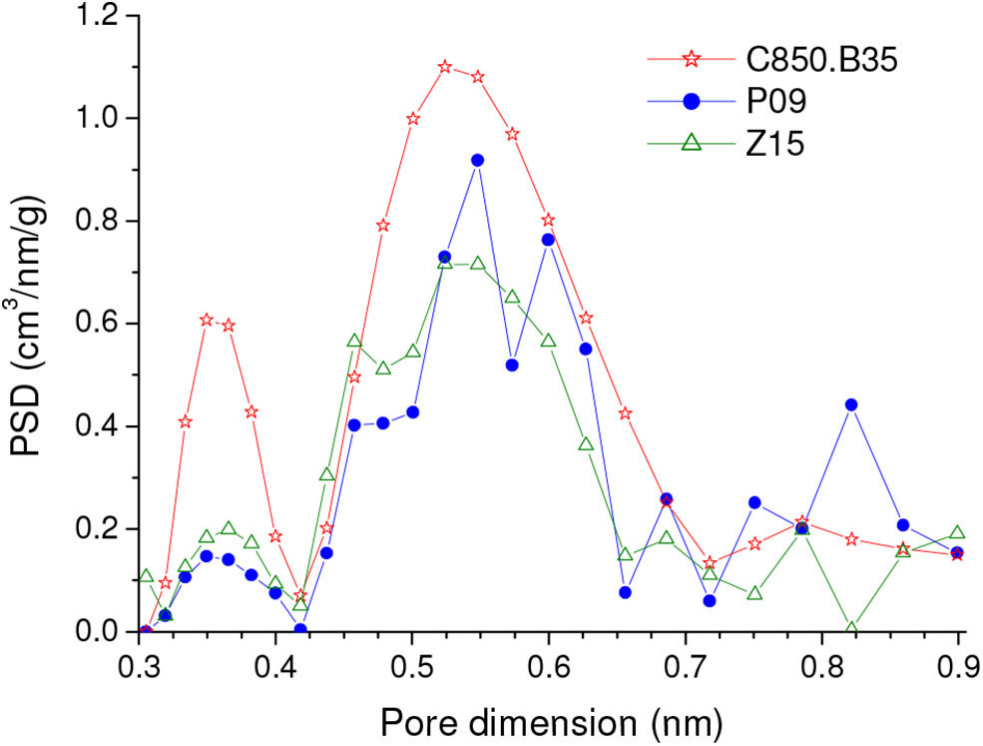
PSDs-CO_2_ curves of representative samples prepared by the different methodologies: physical activation with CO_2_ and chemical activation with H_3_PO_4_ or ZnCl_2_.

The comparison of the results verified for the samples prepared by the different methodologies shows that the carbons physically activated with CO_2_ reached higher CO_2_ uptakes than the chemically activated samples, whether at 1.0 or 0.15 bar, whether on a mass or a volume basis. This behavior is disclosed by Figure 7, in which the highest CO_2_ uptake reached for each series of samples is plotted in a bar graph. Furthermore, in the case of the gravimetric uptake, the Supplementary Figure 1 makes clear that the physically activated carbons (C850.BXX series) exhibited always higher performances than the chemically activated carbons (PXX and ZXX series). These results can be mainly attributed to the higher volume of ultramipores reached by the physically activated carbons in both the lower and higher range of ultramicropores, as portrayed by the PSD-CO_2_ curves in Figure 8. At this point, it is valid to emphasize that the chemical procedures did not permit to increase considerably the volume of ultramicropores because the use of higher chemical loadings leads only to increases in the volumes of supermicropores or even mesopores.

**Figure 9:**
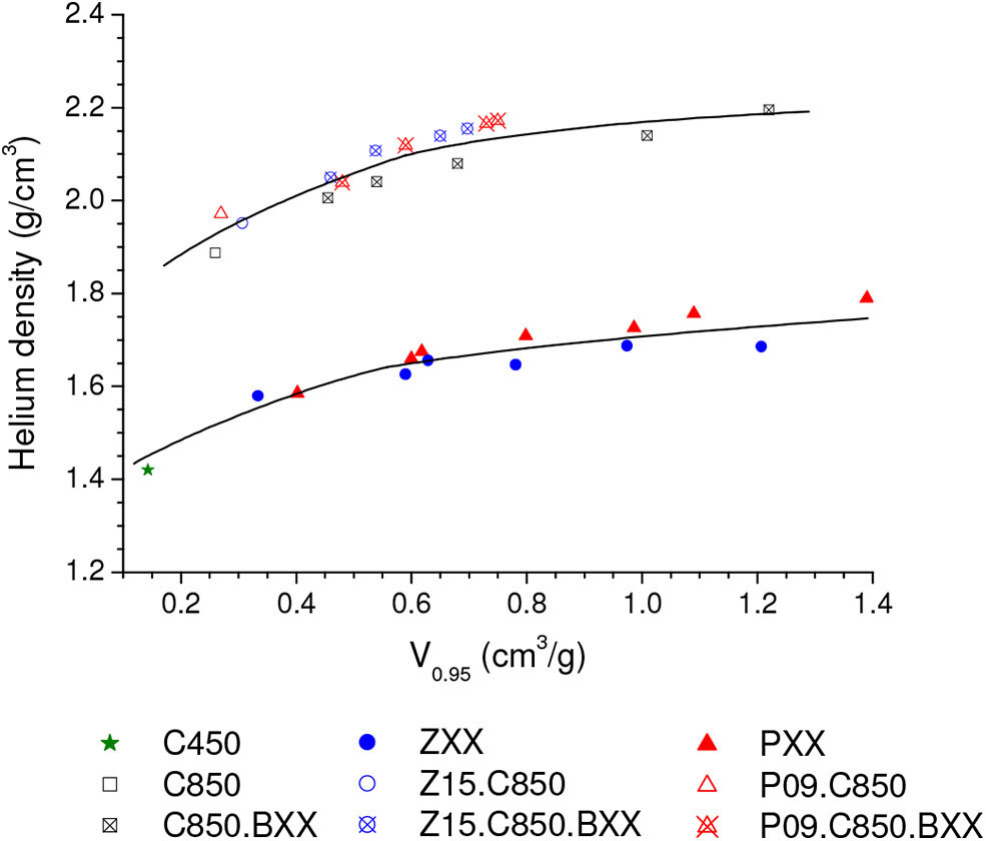
Helium density of all prepared samples as a function of *V*_0.95_.

In the specific case of the volumetric uptake, another factor positively affects the performance of the physically activated carbons: their higher skeleton density. Figure 9 portrays that all the carbons that underwent carbonization at 850°C (as it was the case of the physically activated samples) presented higher helium density than the samples that were treated up to only 450 or 500°C (as it was the case of the chemically activated samples). This occurs because temperatures in the range of ~600–800°C promote extensive aromatization, which increases the skeleton density (Prauchner et al., 2005).

### Complementary Carbonization and Physical Activation of Chemically Activated Carbons

As mentioned in the previous Subsection, the carbons prepared by chemical activation with H_3_PO_4_ or ZnCl_2_ had lower CO_2_ uptakes than those prepared by physical activation with CO_2_. On the other hand, we have shown elsewhere (Prauchner and Rodríguez-Reinoso, 2012; Prauchner et al., 2016) that chemical activation with H_3_PO_4_ or ZnCl_2_ permits to suppress the formation of large macropores that are present in physically activated carbons produced from lignocellulosic feedstocks. These macropores, which correspond to empty spaces that originate from conductor vessels present in the botanical structure of the precursor, few contribute for adsorption and reduce the material bulk density, thus reducing the volumetric adsorption capacity. However, the acid attack promoted by H_3_PO_4_ and ZnCl_2_ degrades the lignocellulosic structure during early carbonization stages, so that the resulting fragments have sufficient mobility to promote a material reorganization and redistribution. As mentioned in the previous Subsection, the reorganization suppresses the formation of small ultramicropores that correspond to free interstices generated during the carbonization process. In turn, the redistribution promotes the filling of existing empty spaces that, otherwise, would result in the formation of macropores.

In this context, we decided to research deeper the feasibility of using chemical activation to prepare ACs with improved volumetric CO_2_ adsorption capacities. We first investigated the possibility of rising the performance of chemically activated carbons by subjecting them to complementary carbonization up to 850°C. For this purpose, we choose the samples activated with the lower chemical loadings (P09 and Z15) because, as already discussed, amongst the chemically activated samples, they presented the more appropriate porosity for CO_2_ adsorption. Afterward, we investigated the possibility of optimizing the porosity of the materials obtained from the complementary carbonization by means of subsequent physical activation with CO_2_.

#### Complementary Carbonization of Chemically Activated Carbons

**Figure 10:**
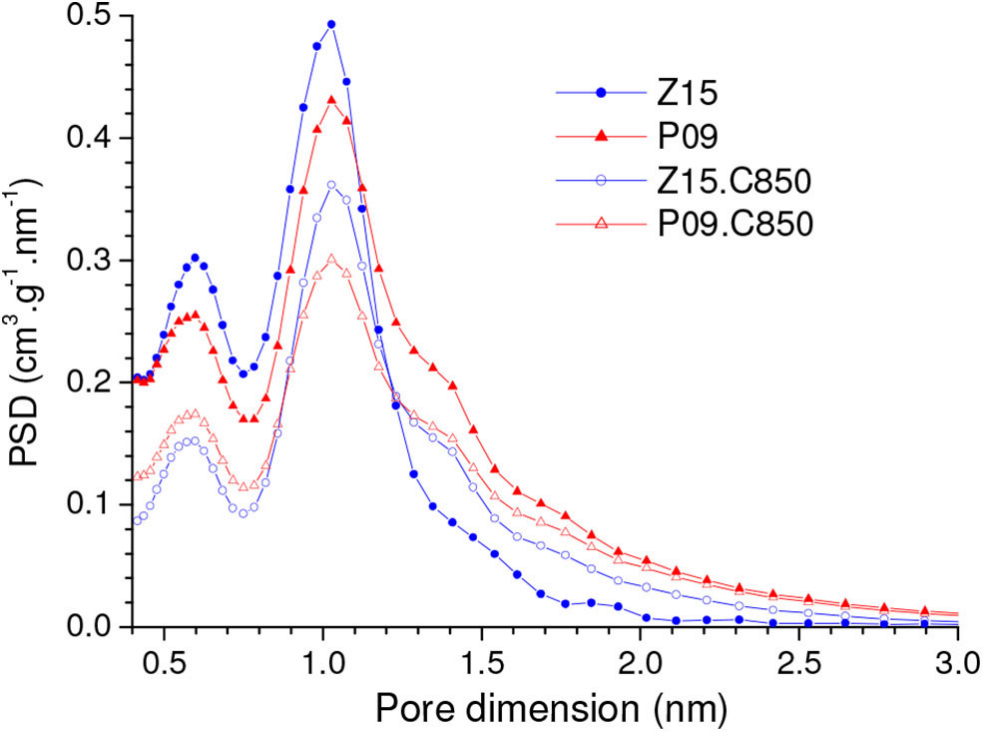
PSD-N_2_ curves of the chemically activated carbons P09 and Z15 and the materials resulting from their complementary carbonization up to 850°C (P09.C850 and Z15.C850, respectively).

**Figure 11:**
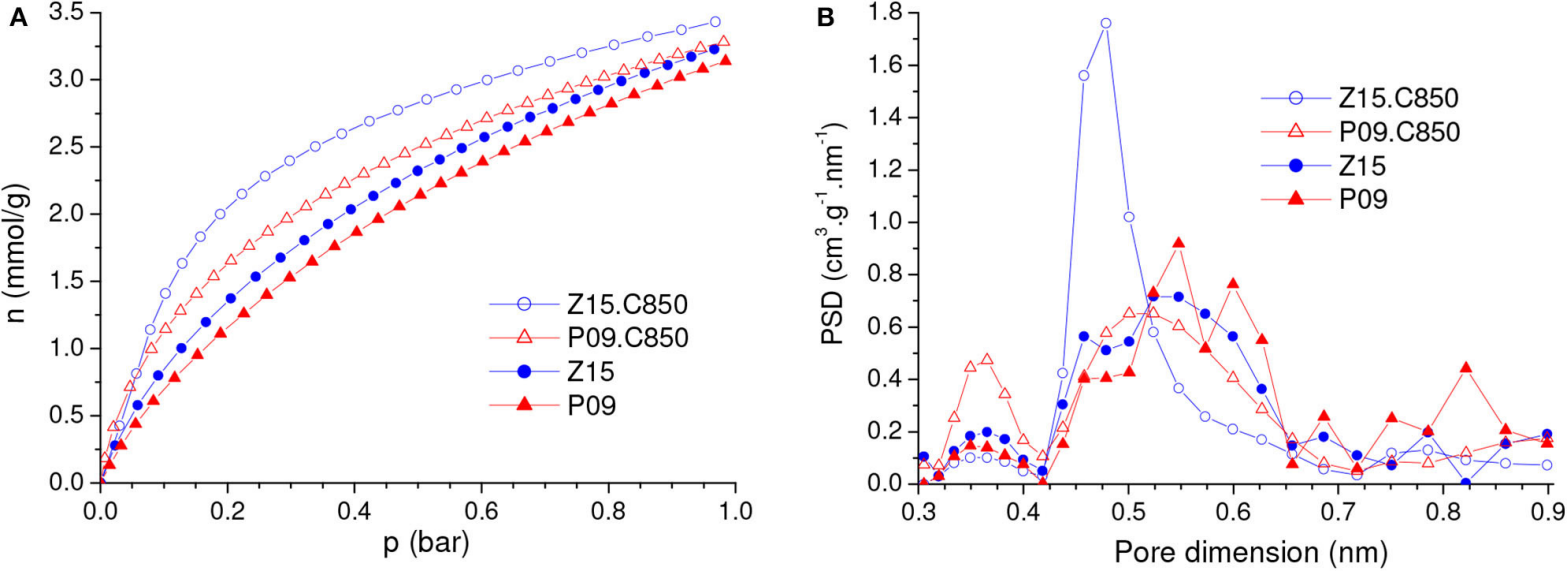
**(A)** CO_2_ adsorption isotherms and corresponding **(B)** PSDs-CO_2_ curves of the chemically activated samples P09 and Z15 and the samples resulting from their complementary carbonization up to 850°C.

Since during chemical activation the materials were treated only up to moderate temperatures (450 or 500°C), complementary carbonization of the P09 and Z15 samples up to 850°C led to additional weight loss of around 8%. This weight loss shrank the particles, thus causing porosity contraction. Therefore, there was considerable reduction in the volume of pores that were accessible to N_2_ at −196°C. As disclosed by the PSD-N_2_ curves in Figure 10, these pores were mostly situated in the range of supermicropores (0.7 nm < d < 2.0 nm). Furthermore, there was also some reduction in the volume of pores located in the higher range of ultramicropores, as portrayed by the PSD-CO_2_ curves in Figure 11B. On the other hand, this same Figure evidences that the contraction of larger pores ended by increasing the volume of pores located in the lower range of ultramicropores. This effect was remarkably pronounced in the case of the Z15.C850 sample, whose PSD-CO_2_ curve exhibited a somewhat sharp peak centered at around 0.47 nm.

The comparison of the CO_2_ adsorption isotherms of the Z15.C850 and P09.C850 samples with those of the Z15 and P09 samples (Figure 11A) reveals that complementary carbonization increased the gravimetric CO_2_ uptake. In line with the changes in porosity reported in the previous paragraph, the increases were more pronounced at lower pressures: while at 1.0 bar the uptake increased only from 3.18 and 3.28 mmol/g for the P09 and Z15 samples to 3.31 and 3.48 mmol/g for the P09.C850 and Z15.C850 samples (increases of around 4 and 8%, respectively), at 0.15 bar the increases were much higher, from 0.95 and 1.11 mmol/g to 1.40 and 1.78 mmol/g (increases of around 47 and 60%, respectively).

Besides increasing the gravimetric CO_2_ uptake, complementary carbonization considerably increased the bulk density: the increases were from 0.536 and 0.544 g/cm^3^ for the P09 and Z15 samples to 0.680 and 0.660 g/cm^3^ for the P09.C850 and Z15.C850 samples (Table 1). This increase can be attributed to two effects: (i) the porosity contraction; (ii) the increase of skeleton density (see Figure 9 and related discussion).

Since both the gravimetric CO_2_ uptake and bulk density increased with complementary carbonization, the volumetric uptake also increased: at 1.0 bar, it increased from 1.70 and 1.78 mmol/cm^3^ for the P09 and Z15 samples to 2.25 and 2.30 mmol/cm^3^ for the P09.C850 and Z15.C850 samples; at 0.15 bar, the respective increases were from 0.51 and 0.60 mmol/cm^3^ to 0.95 and 1.17 mmol/cm^3^.

#### Physical Activation of Chemically Activated Carbons

**Figure 12:**
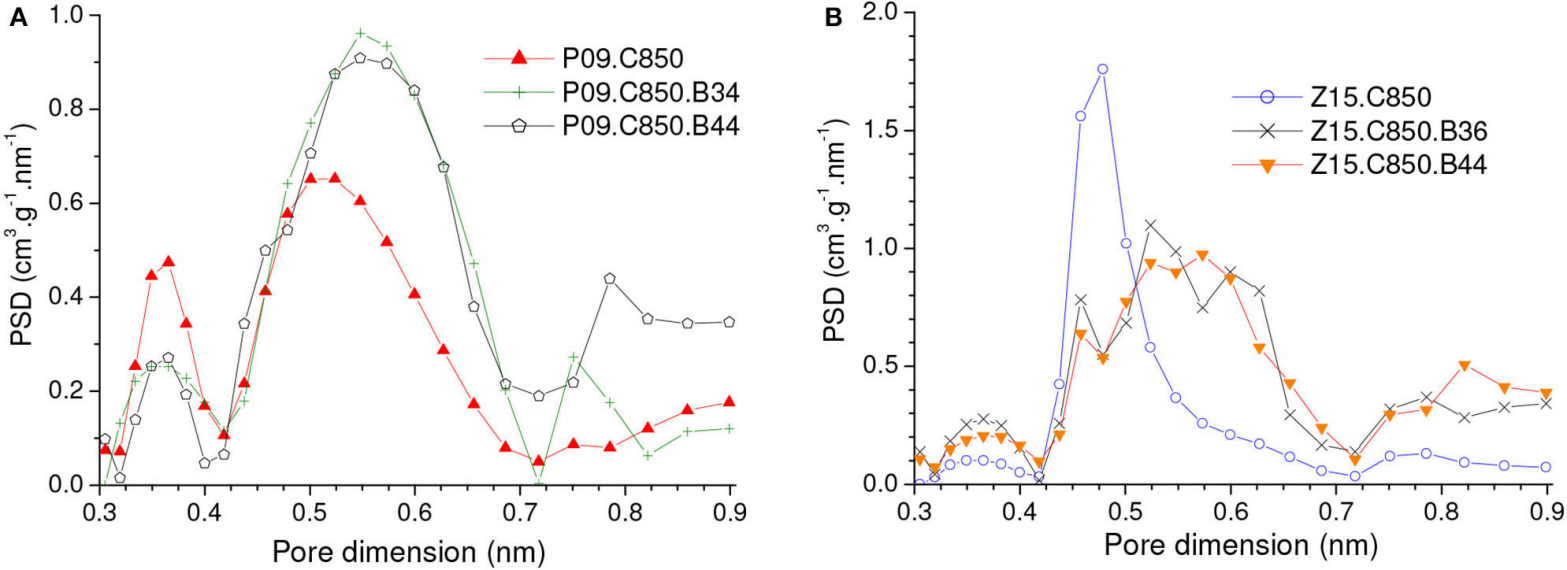
PSDs-CO_2_ curves of carbons produced through complementary carbonization of the chemically activated samples **(A)** P09 and **(B)** Z15, besides some selected samples obtained by subsequent physical activation with CO_2_.

**Figure 13:**
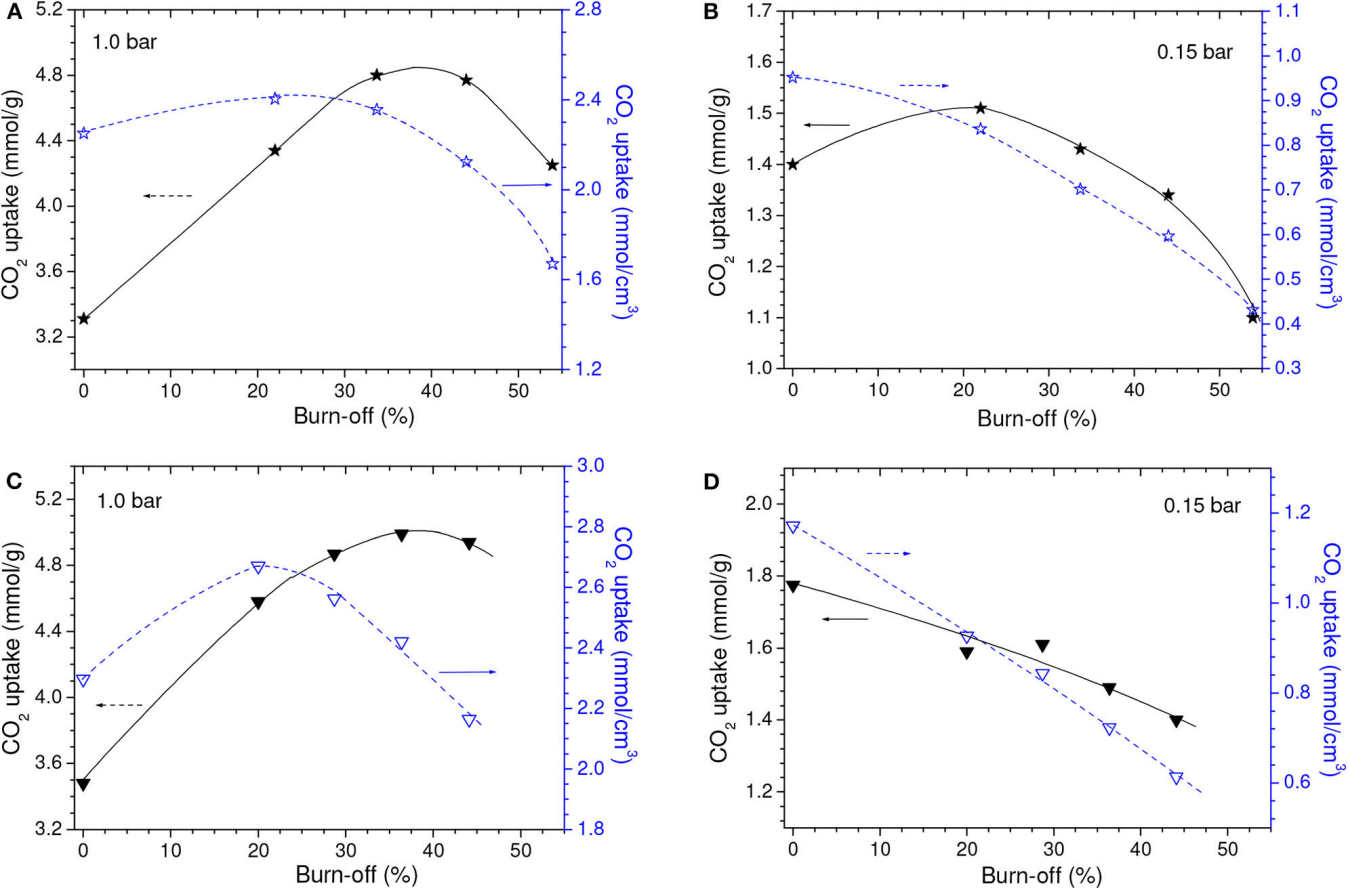
Gravimetric and volumetric CO_2_ uptakes at **(A,C)** 1.0 and **(B,D)** 0.15 bar as a function of the burn-off during physical activation of the **(A,B)** P09.C850 and **(C,D)** Z15.C850 samples.

As disclosed by the *V*_0.95_ values in Table 1, physical activation with CO_2_ permitted to develop the porosity of the P09.C850 and Z15.C850 samples. Regarding the ultramicropores, Figure 12 shows that the volume of pores in the higher range of ultramicropores (higher than nearly 0.5 nm) pronouncedly increased up to a burn-off of around 35% and, from that point on, it stopped to increase and even slightly diminished. Therefore, the gravimetric CO_2_ uptake at 1.0 bar passed through a maximum at a burn-off of nearly 35% and decreased thereafter. The maxima were ~4.80 and 4.99 mmol/g for the P09.C850.BXX and Z15.C850.BXX series, respectively (Figures 13A,C).

Maximum values were also observed for the volumetric CO_2_ uptake at 1.0 bar (Figures 13A,C). However, thanks to the effect of the decreasing bulk density, the burn-off required to achieve the maximum volumetric CO_2_ uptake at 1.0 bar (~20%) was lower than that needed to attain the highest gravimetric value (~35%). This phenomenon shows that, even though the volume of ultramicropores and the gravimetric CO_2_ uptake keep increasing throughout the burn-off range of around 20–35%, the density of the adsorbed phase diminishes, so that the volumetric uptake decreases. In the case of the P09.C850.BXX series, the maximum (2.40 mmol/cm^3^ for the P09.C850.B22 sample) was only slightly higher than that of the correspondent non-gasified sample P09.C850 (2.25 mmol/cm^3^). However, in the case of the Z15.C850.BXX series, significant gain was achieved: the volumetric uptake increased from 2.30 mmol/cm^3^ for the non-gasified carbon Z15.C850 to 2.67 mmol/cm^3^ for the Z15.C850.B20 sample. This result can be attributed to the already mentioned narrow size distribution of ultramicropores presented by the Z15.C850 sample, which could be carefully tailored by gasification in order to render an AC with optimized pore size distribution for CO_2_ adsorption.

**Figure 14:**
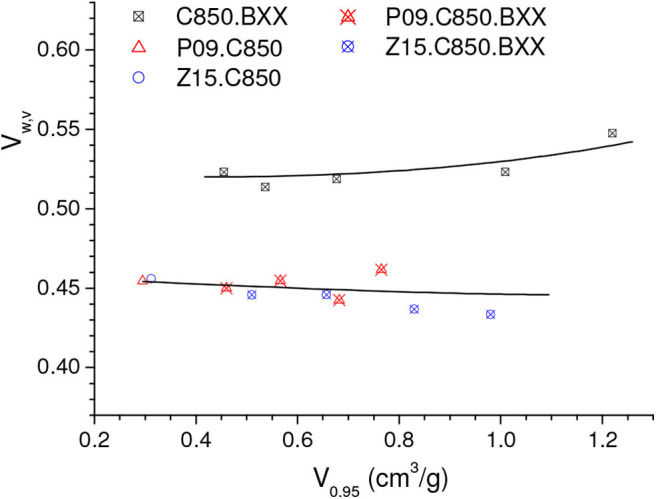
Volumetric waste volume (*V*_*w,v*_) as a function of *V*_0.95_.

The volumetric uptake at 1.0 bar verified for the Z15.C850.B20 sample (2.67 mmol/cm^3^) was considerably higher than that observed for the C850.B25 sample (2.34 mmol/g), the one that presented the maximum value in the series prepared by physical activation of the carbons that were not submitted to a previous chemical activation. However, it is interesting to note the Z15.C850.B20 sample presented an even somewhat lower gravimetric uptake at 1.0 bar (4.58 mmol/g) than the C850.B25 sample (4.68 mmol/g), so that the higher volumetric uptake of the former can be attributed to its higher bulk density. In turn, since the samples Z15.C850.B20 and C850.B25 presented similar *V*_0.95_ (0.464 and 0.455 cm^3^/g, respectively) and helium density (2.06 and 2.04 g/cm^3^), thus it is possible to infer that the higher bulk density of the Z15.C850.B20 sample is due to its lower waste volume. Indeed, Figure 14 shows that the samples that underwent chemical activation with ZnCl_2_ or H_3_PO_4_ (those pertaining to the PXX, ZXX, P09.C850.BXX and Z15.C850.BXX series, besides the samples P09.C850 and Z15.C850) had lower waste volume than the samples that were heat treated only in absence of chemicals (samples pertaining to the C.850.BXX series, besides the samples C450 and C850). As already discussed along the text, such lower waste volume can be attributed to the action of the chemical during the early stages of carbonization, which prevents the occurrence of macropores.

If by one hand the gasification with CO_2_ increased the volume of pores in the higher range of ultramicropores, on the other hand it decrease the volume of pores in the lower range (Figure 12). In the case of the samples that were previously chemically activated with H_3_PO_4_ (P09.C850.BXX series), this decrease was restricted to the pores smaller than ~0.4 nm, so that it did not considerably affect the capacity of adsorbing CO_2_. Therefore, the gravimetric CO_2_ uptake at 0.15 bar somewhat increased with low burn-offs, being that a maximum value was verified for the P09.C850.B22 sample (0.88 mmol/cm^3^; Figure 13B). In turn, in the case of the samples that were previously chemically activated with ZnCl_2_, the volume of pores pronouncedly decreased in the range of nearly 0.42–0.51 nm. Thus, all ACs pertaining to the Z15.C850.BXX series had gravimetric CO_2_ uptake lower than the corresponding non-gasified Z15.C850 sample (Figure 13D).

Thanks to the results verified for the gravimetric CO_2_ uptake at 0.15 bar and the fact that the bulk density decreased with increasing burn-off, all the gasified samples pertaining to the P09.C850.BXX and Z15.C850.BXX series had lower volumetric CO_2_ uptake than the corresponding non-gasified P09.C850 and Z15.C850 samples. Furthermore, the volumetric uptake steadily decreased with increasing burn-off.

### Final Considerations

The highest volumetric CO_2_ uptakes attained in the present work were 2.67 and 1.17 mmol/cm^3^ for CO_2_ pressures of 1.0 and 0.15 bar. These values were verified for the Z15.C850.B20 and Z15.C850 samples, respectively. It is noteworthy that the preparation of both these samples involved a preliminary chemical activation with a low ZnCl_2_ loading, followed by complementary carbonization up to 850°C. In the case of the sample Z15.C850.B20, a soft gasification with CO_2_ was also employed. These results and the discussions along the text permitted to make important insights about the way the processing methodology influences the characteristics of the resulting adsorbents and their performance for CO_2_ adsorption. Firstly, a chemical activation step (with H_3_PO_4_ or ZnCl_2_) permits to suppress the occurrence of macropores, what is beneficial for the bulk density. Nevertheless, it is needed to mention that a low chemical loading must be used because, otherwise, the pores widen and, therefore, become less efficient for CO_2_ adsorption at low pressures. Secondly, it is important to submit the material to a subsequent thermal treatment up to relatively high temperatures (like 850°C) in order to increase the material skeleton density and reduce the average size of the pores created by chemical activation. Finally, if compared with H_3_PO_4_, ZnCl_2_ is a more appropriate chemical agent because it renders a more homogeneous distribution of ultramicropores. After carbonization at 850°C, these pores become appropriate for CO_2_ adsorption at low subatmospheric pressures (e.g., 0.15 bar). In turn, for adsorption at atmospheric pressure, a subsequent soft gasification with CO_2_ makes it possible to enhance the pores in such a controlled way that improved volumetric CO_2_ uptakes can be attained.

If taken on a gravimetric basis, the CO_2_ uptakes obtained in the present work were lower than those usually reported in the literature for ACs prepared by chemical activation with KOH. For example, the highest values we attained at 1.0 and 0.15 bar were ~5.00 and 2.00 mmol/g, respectively, whereas the corresponding values reported by Li et al. (2019) for petroleum coke-based ACs were 6.47 and 2.52 mmol/g. Notwithstanding, if a volumetric basis is taken into account, the differences disappear. Namely, the highest volumetric CO_2_ uptakes verified in the present work, 2.67 and 1.17 mmol/cm^3^, were quite similar to those verified by the team of Li, 2.64 and 1.18 mmol/cm^3^ (the authors considered the bulk density as the tap density measured for the powdered AC with particle size 50–74 μm).

At this point, it is worthy to highlight that the adsorbents prepared by Li et al. (2019) were chemically activated with a high proportion of a caustic and corrosive chemical agent (the employed KOH:precursor ratio was 1.5), whereas the samples that displayed the highest volumetric uptakes in our work were activated with a softer chemical, ZnCl_2_, with a Zn:precursor ratio of only 0.15. Further, the ACs prepared by the team of Li were in the form of a fine powder, which is unpractical for fixed bed adsorption columns, whereas our ACs were prepared as grains with high mechanical resistance, which makes them very appropriate adsorbents for this kind of application.

In addition, it is also valid to mention that the interparticle space of granular carbons can be reduced by providing and adequate mix of samples with different granulometries, in which smaller grains partially occupy the free space among the larger grains (Prauchner and Rodríguez-Reinoso, 2008). Therefore, we foresee that it is possible to improve by around 8–10% the maximum volumetric adsorption capacities we reported in the present work.

As already mentioned in the Introduction Section, there are in the literature three other papers that reported the CO_2_ adsorption capacity of ACs on a volumetric basis, the papers of Silvestre-Albero et al. (2011), Marco-Lozar et al. (2012), and Haffner-Staton et al. (2016). However, the comparison of the results attained in our work with those reported by these authors is hampered because they: (i) measured the CO_2_ uptake at a different temperature, 25°C; (ii) used different approaches to determine the bulk volume. For example, the teams of Silvestre-Albero and Haffner-Staton measured the bulk volume by compacting the powered ACs at high pressures, what does not correspond to a realistic situation at an operating fixed bed adsorption column. In turn, Marco-Lozar et al. considered the bulk volume as the geometric volume of the monoliths. However, the resulting values cannot also be considered realistic because it is unfeasible to have a large scale adsorption column completely filled by monoliths, because the gas diffusion would be precluded. Therefore, it is possible to state that in both works the authors overestimated the bulk densities and, therefore, the volumetric adsorption capacities.

## Conclusions

Granular ACs intended for CO_2_ capture at atmospheric and subatmospheric pressures were prepared from an abundant and low-cost biomass residue by using practical and cost-effective procedures, which is essential for large scale applications such as CO_2_ capture and biogas upgrading. By the first time, parameters involved in the chemical activation with dehydrating agents (H_3_PO_4_ or ZnCl_2_) and/or physical activation with CO_2_ were systematically screened in depth in order to obtain materials with improved performance for CO_2_ capture. The prepared ACs exhibited a blend of relatively high gravimetric CO_2_ uptake and bulk density, so that high volumetric adsorption capacities were attained. The highest values were 2.67 and 1.17 mmol/cm^3^ for CO_2_ pressures of 1.0 and 0.15 bar, respectively. They were verified for the samples that were first chemically activated with ZnCl_2_, followed by carbonization up to 850°C; in the case of the pressure of 1.0 bar, a subsequent soft gasification with CO_2_ was also employed to optimize the pore size distribution.

Remarkably, these results were similar to those reported by other authors for carbons chemically activated with KOH, the activation methodology that has been widely claimed as the one that produce ACs with the best performances for CO_2_ adsorption, but which involves severe restrictions such as the need of using very large proportions of a caustic and corrosive chemical and the fact that the ACs are obtained in the powdered form. Furthermore, it is possible to foresee that the volumetric results reported in the present work can be additionally improved by using a mix of samples with different granulometries, which would permit to reduce the interparticle space. Therefore, the present work can be considered a very important step in paving the way toward making CO_2_ adorption an each time more interesting technology to reduce the emissions of anthropogenic greenhouse gases.

## Data Availability Statement

The original contributions presented in the study are included in the article/Supplementary Materials, further inquiries can be directed to the corresponding author/s.

## Author Contributions

MP and FR-R conceived the study. MP developed most of the experimental work and wrote the first draft of the manuscript. SO assisted MP in data treatment and manuscript writing. All authors read and approved the final manuscript.

## A Tribute to Francisco Rodríguez-Reinoso (1941-2020)

Some time after the submission of this paper, we were shocked about the death of Professor Francisco Rodríguez-Reinoso (on August 25th). I am not able to describe the profound sadness that the news provoked in me. He was a brilliant scientist in the area of activated carbons, with a world-wide reputation. However, much beyond a great scientist, professor Rodríguez-Reinoso was also a great person, a precious friend and a great family man. His teachings will certainly remain forever, influencing all those who, like me, had the privilege of having him as professor, supervisor and friend. Dear professor Rodríguez-Reinoso, thank you very much for all you have provided to me and other hundreds of scientists all over the world.

## Conflict of Interest

The authors declare that the research was conducted in the absence of any commercial or financial relationships that could be construed as a potential conflict of interest.
