# Supplementary material for: Tailoring Low-Cost Granular Activated Carbons Intended for CO2 Adsorption
Source: Front Chem. 2020 Nov 19;8:581133. doi: 10.3389/fchem.2020.581133 (PMC7718001; doi:10.3389/fchem.2020.581133)
Supplement: Supplementary file 1 [file Data_Sheet_1.PDF]

Supplementary Material

Supplementary Table 1. Gravimetric CO_2 uptake (Q_g) at 1.0 bar and 0 °C of some samples selected from the literature (as a general rule, the highest value reported in each work was taken).

Sample	Precursor	Activating agent	Q_g (mmol/g)	Reference[a]
-	Pine nut shell	KOH	7.7	Deng et al., 2014
VR-5-M	Petroleum pitch	KOH	8.6	Wahby et al., 2010
PANI_C400_KC650	Polyaniline	KOH	7.6	Silvestre-Albero et al., 2014
C600K3	Palm shell	KOH	6.3	Ello et al., 2013a
AS-2-600	Sawdust	KOH	6.1	Sevilla and Fuertes, 2011
PC3-780	Rice husk	KOH	6.2	Li et al., 2015
LAC2700	Lignin waste	KOH	7.4	Sangchoom and Mokaya, 2015
AC-680	Petroleum coke	KOH	6.5	Li et al., 2019a
MCC-K3	Wheat	KOH	5.7	Hong et al., 2016
AC-26	Garlic peel	KOH	6.3	Huang et al., 2019
800_1.5	Lumpy bracket	KOH	7.0	Serafin et al., 2019
ACDS-800-4	Date sheet	KOH	6.4	Li et al., 2019b
C500-K	Petroleum coke	KOH	6.7	Jang et al., 2019
P2_3	Polyaniline	KOH	9.14	Kutorglo et al., 2019
H150–800/H250–800	Empty fruit bunch	KOH	5.2	Parshetti et al., 2015
Bamboo-3-873	Bamboo	KOH	7.0	Wei et al., 2012
NPC-650	Polyimine	KOH	5.3	Wang et al., 2013
C-KOH/C-CO_2	Commercial carbon Ambersorb 56	KOH/ CO_2	6.9/5.5	Ludwinowicz and Jaroniec, 2015
cnut 3.5h	Coconut shell	CO_2	5.6	Ello et al., 2013b
A	Macadamia nut shell	CO_2	4.6	Bae and Su, 2013
AC-2/PC-AC-1	Slash pine	KOH/$ZnCl_2$	4.9/4.5	Ahmed et al., 2019
AC60	Olive stone	$ZnCl_2$ + CO_2	4.8	Balsamo et al., 2014
CACM32/ PACM28	coconut/palm stones	H_3PO_4	3.7/3.7	Vargas et al., 2011
MSS-AC	Mango seed shells	H_3PO_4	4.8	Munusamy et al., 2015

[a]The references cited here are listed at the end of this Supplementary Material

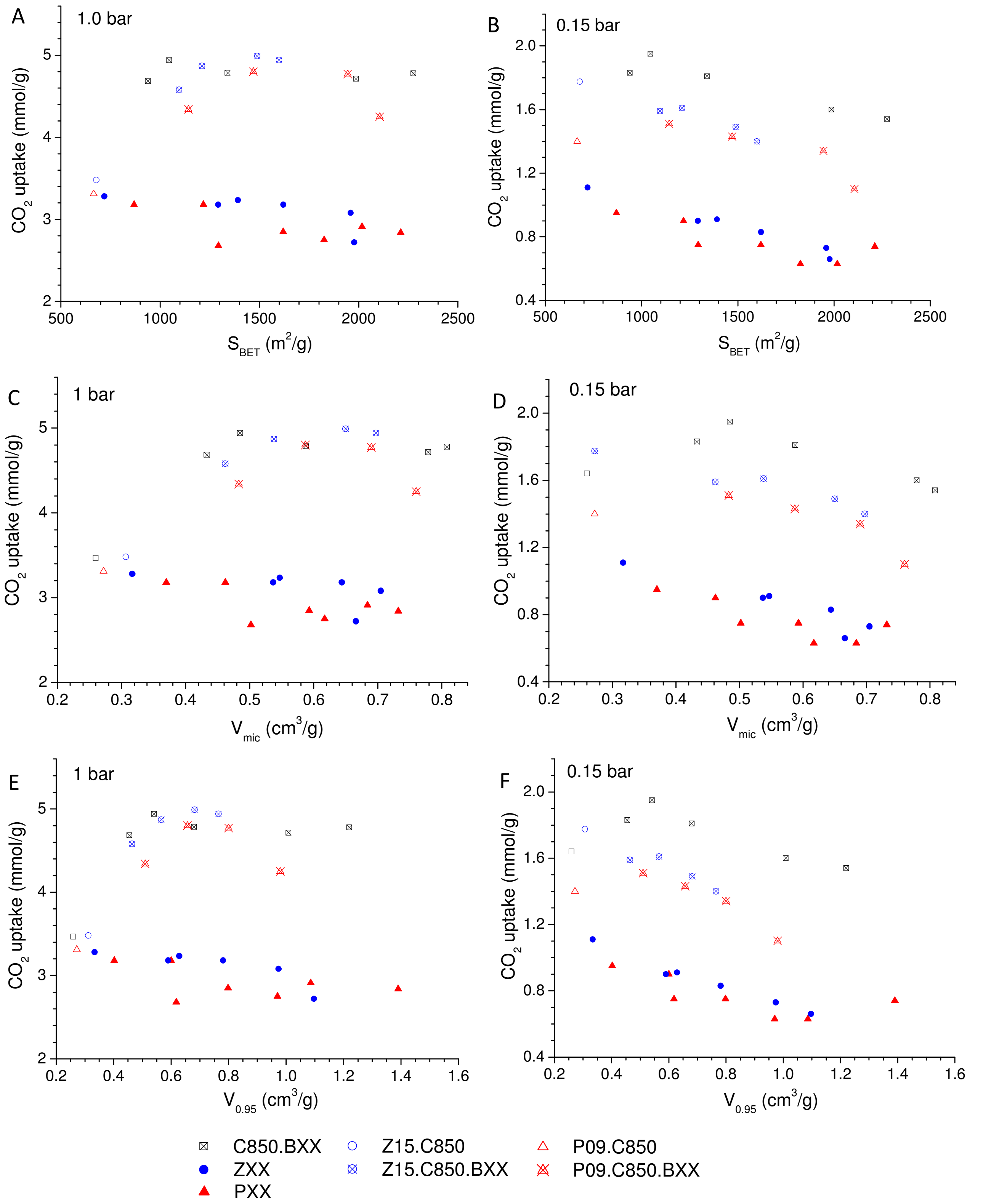

Supplementary Figure 1. Plots of gravimetric CO_2 uptake at (A, C, E) 1.0 bar and (B, D, F) 0.15 bar as a function of S_{BET}, V_{mic} and $V_{0.95}$.

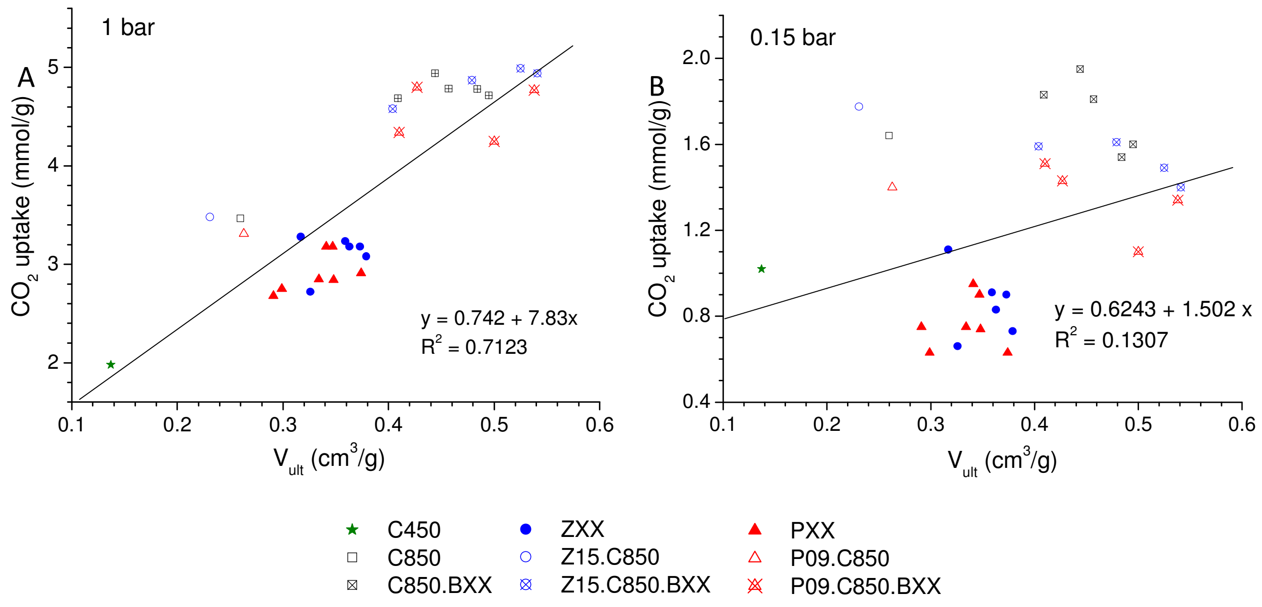

Supplementary Figure 2. Plots of gravimetric CO_2 uptake at (A) 1.0 bar and (B) 0.15 bar as a function of V_{ult}.

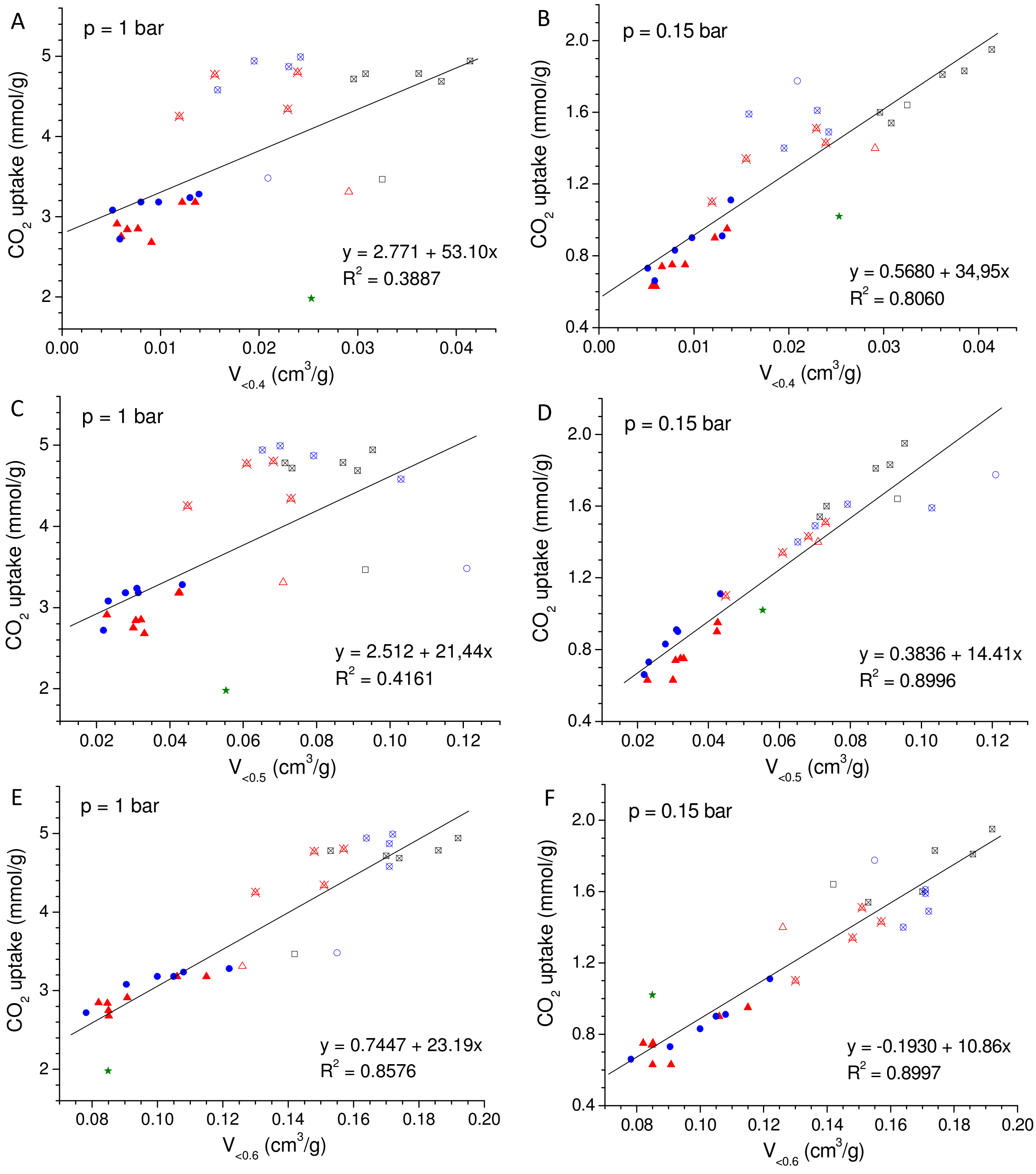

Supplementary Figure 3 (it continues in the next page).

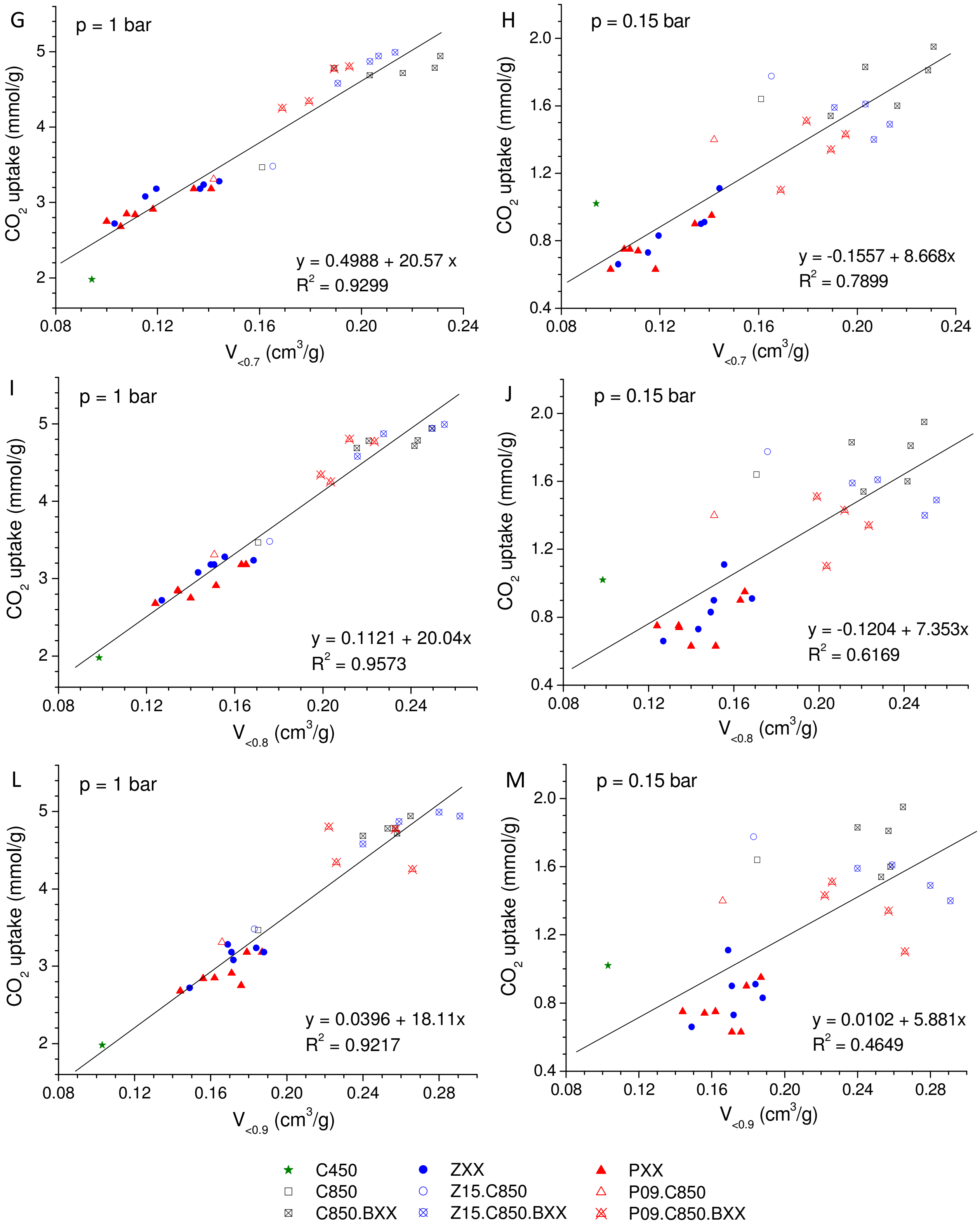

Supplementary Figure 3. Plots of gravimetric CO_2 uptake at (A, C, E, G, I, L) 1 bar and (B, D, F, H, J, M) 0.15 bar as a function of $V_{<0.4}$, $V_{<0.5}$, $V_{<0.6}$, $V_{<0.7}$, $V_{<0.8}$ and $V_{<0.9}$.

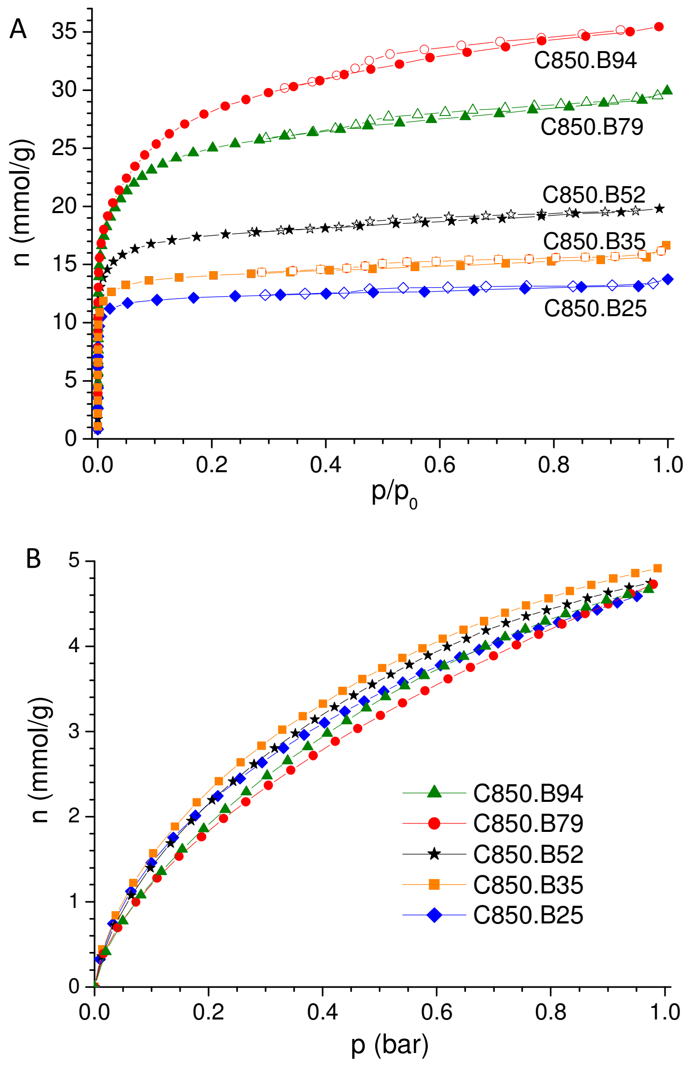

Supplementary Figure 4. (A) N_2 adsorption-desorption and (B) CO_2 adsorption isotherms of the samples physically activated with CO_2. Closed symbols: adsorption; open symbols: desorption.

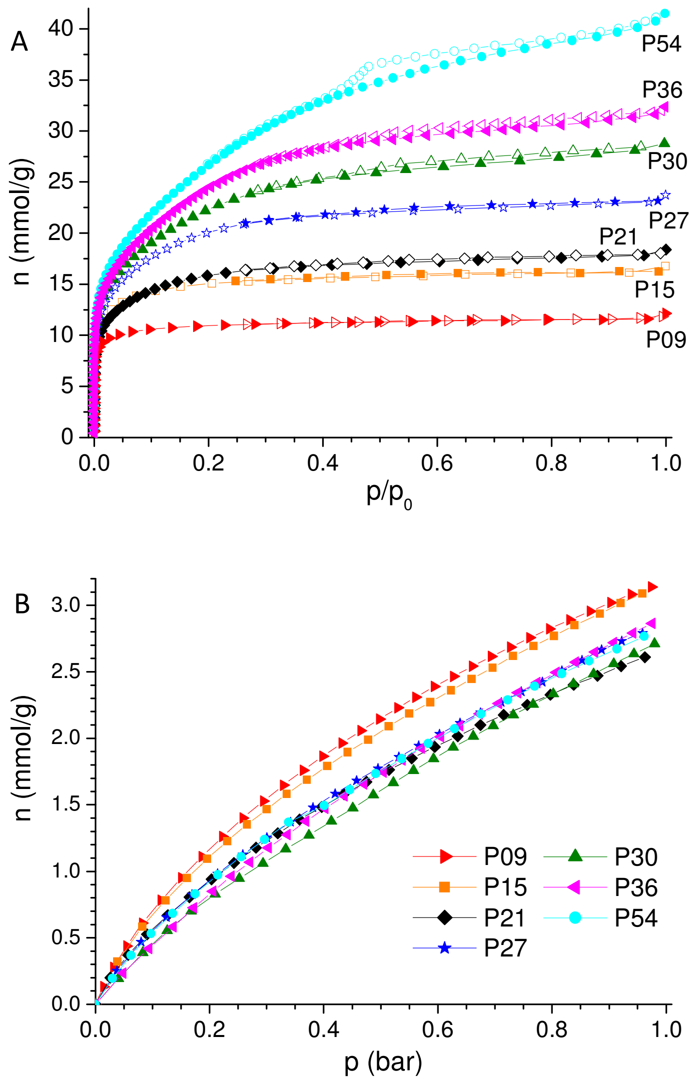

Supplementary Figure 5. (A) N_2 adsorption-desorption and (B) CO_2 adsorption isotherms of the samples chemically activated with H_3PO_4. Closed symbols: adsorption; open symbols: desorption.

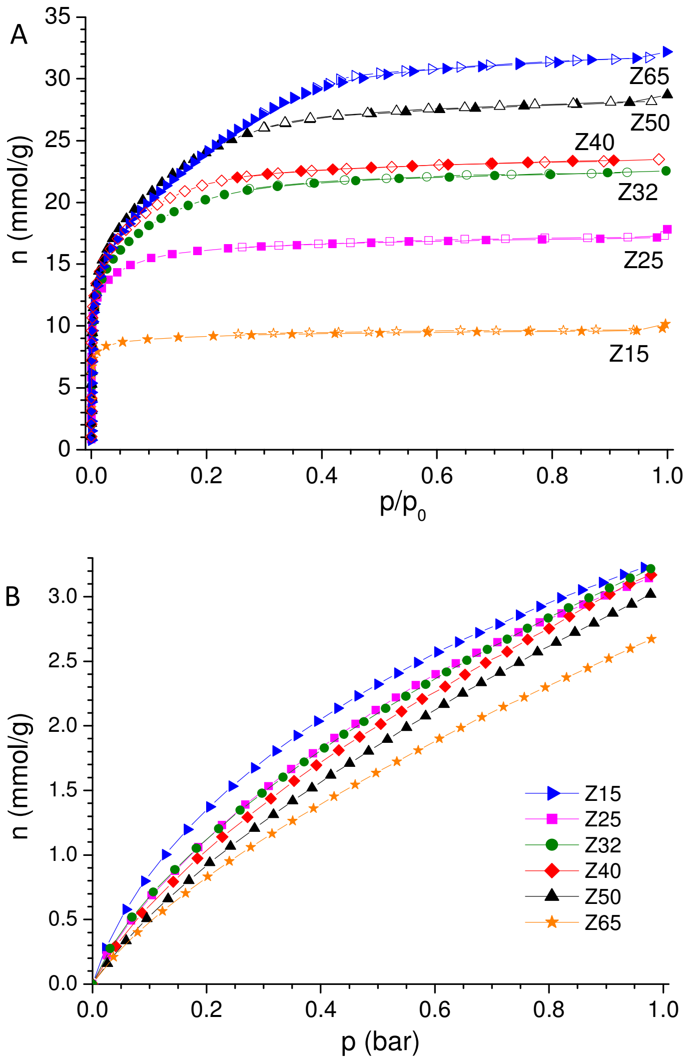

Supplementary Figure 6. (A) N_2 adsorption-desorption and (B) CO_2 adsorption isotherms of the samples chemically activated with $ZlCl_2$. Closed symbols: adsorption; open symbols: desorption.

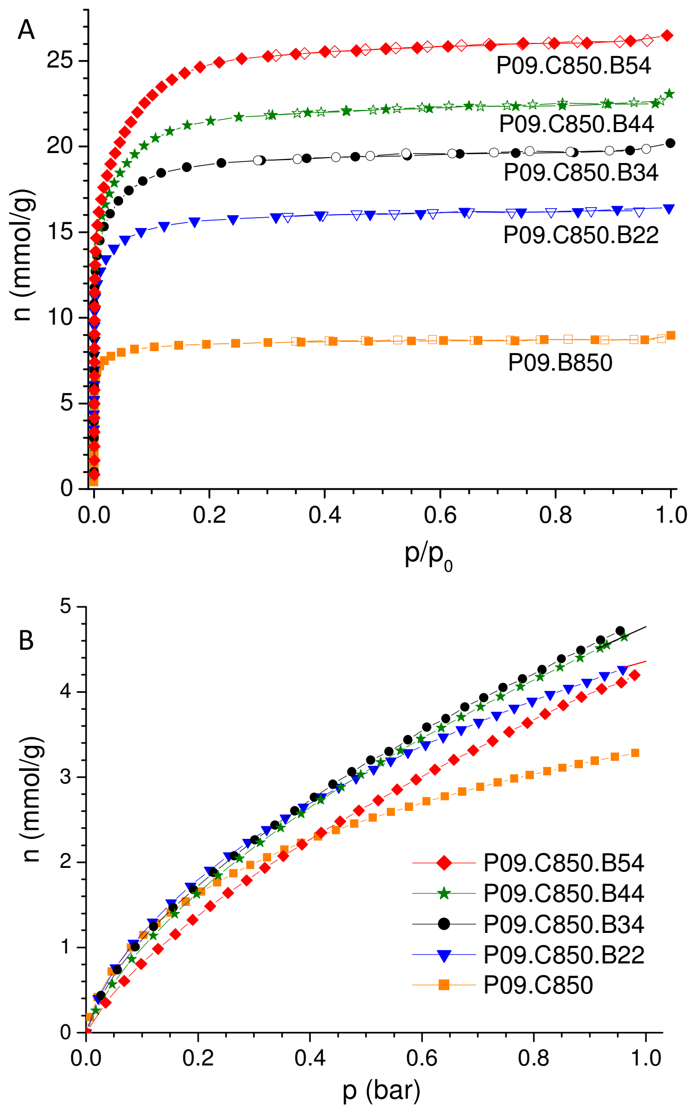

Supplementary Figure 7. (A) N_2 adsorption-desorption and (B) CO_2 adsorption isotherms of the sample P09.C850 and the carbons resulting from its subsequent physical activation with CO_2. Closed symbols: adsorption; open symbols: desorption.

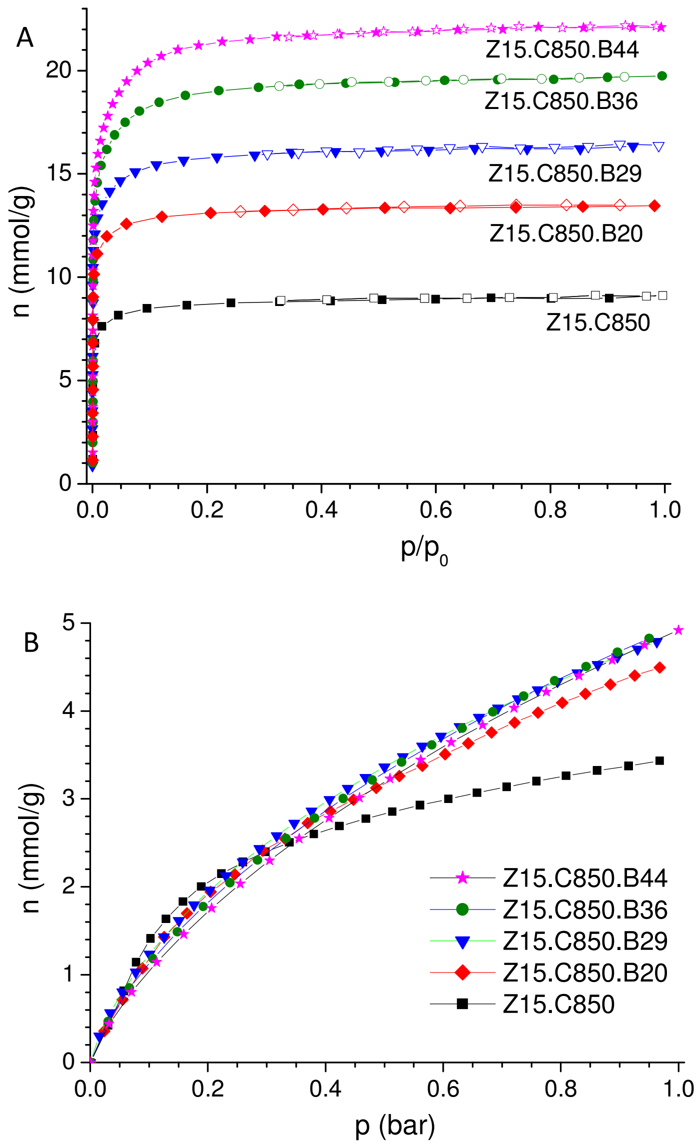

Supplementary Figure 8. (A) N_2 adsorption-desorption and (B) CO_2 adsorption isotherms of the sample Z15.C850 and the carbons resulting from its subsequent physical activation with CO_2. Closed symbols: adsorption; open symbols: desorption.

Supplementary references

Ahmed, M. B., Johir, M. A. H., Zhou, J. L., Ngo, H. H., Nghiem, L. D., Richardson, C., et al. (2019). Activated carbon preparation from biomass feedstock: clean production and carbon dioxide adsorption. J. Clean. Prod. 225, 405–413. doi: 10.1016/j.jclepro.2019.03.342

Bae, J.-S., and Su, S. (2013). Macadamia nut shell-derived carbon composites for post combustion CO_2 capture. Int. J. Greenhouse Gas Control 19, 174–182. doi: 10.1016/j.ijggc.2013.08.013

Balsamo, M., Silvestre-Albero, A., Silvestre-Albero, J., Erto, A., Rodriguez-Reinoso, F., and Lancia, A. (2014). Assessment of CO_2 adsorption capacity on activated carbons by a combination of batch and dynamic tests. Langmuir 30, 5840–5848. doi: 10.1021/la500780h

Deng, S., Wei, H., Chen, T., Wang, B., Huang, J., and Yu, G. (2014). Superior CO_2 adsorption on pine nut shell-derived activated carbons and the effective micropores at different temperatures, Chem. Eng. J. 253, 46–54. doi: 10.1016/j.cej.2014.04.115

Ello, A. S., de Souza, L. C. K., Trocourney, A., and Jaroniec, M. (2013a). Development of microporous carbons for CO_2 capture by KOH activation of African palm shells. J. CO_2 Util. 2, 35–38. doi: 10.1016/j.jcou.2013.07.003

Ello, A. S., Souza, L. C. K., Trocourney, A., and Jaroniec, M. (2013b). Coconut shell-based microporous carbons for CO_2 capture. Micropor. Mesopor. Mat. 180, 280–283. doi: 10.1016/j.micromeso.2013.07.008

Hong, S. M., Jang, E., Dysart, A. D., Pol, V. G., and Lee, K. B. (2016). CO_2 capture in the sustainable wheat-derived activated microporous carbon compartments. Sci. Rep. 6, 34590–34600. doi: 10.1038/srep34590

Huang, G-G., Liu,Y-F, Wu, X-X., and Cai, J-J. (2019). Activated carbons prepared by the KOH activation of a hydrochar from garlic peel and their CO_2 adsorption performance. New Carbon Mater. 34, 247–257. doi: 10.1016/S1872-5805(19)60014-4

Jang, E. Choi, S. W., and Lee, K. B. (2019). Effect of carbonization temperature on the physical properties and CO_2 adsorption behavior of petroleum coke-derived porous carbon. Fuel 248, 85–92. doi: 10.1016/j.fuel.2019.03.051

Kutorglo, E. M., Hassouna, F., Beltzung, A., Kopecký, D., Sedlářová, I., and Šoóš, M. (2019). Nitrogen-rich hierarchically porous polyaniline-based adsorbents for carbon dioxide (CO_2) capture. Chem. Eng. J. 360, 1199–1212. doi: 10.1016/j.cej.2018.10.13.

Li, D., Ma, T., Zhang, R., Tian, Y., and Qiao, Y. (2015). Preparation of porous carbons with high low-pressure CO_2 uptake by KOH activation of rice husk char. Fuel 139, 68–70. Doi: 10.1016/j.fuel.2014.08.027

Li, D., Zhou, J., Wang, Y., Tian, Y., Wei, L., Zhang, Z., et al. (2019a). Effects of activation temperature on densities and volumetric CO_2 adsorption performance of alkali-activated carbons. Fuel 238, 232–239. doi:10.1016/j.fuel.2018.10.122

Li, J., Michalkiewicz, B., Min, J., Ma, C., Chen, X., Gong, J., et al. (2019b). Selective preparation of biomass-derived porous carbon with controllable pore sizes toward highly efficient CO_2 capture. Chem. Eng. J. 360, 250–259. doi: 10.1016/j.cej.2018.11.204

Ludwinowicz, J., and Jaroniec M. (2015). Effect of activating agents on the development of microporosity in polymeric-based carbon for CO_2 adsorption. Carbon 94, 673–679. doi: 10.1016/j.carbon.2015.07.052

Munusamy, K., Somani, R. S., and Bajaj H. C (2015). Breakthrough adsorption studies of mixed gases on mango (Mangifera indica L.) seed shell derived activated carbon extrudes, J. Environ. Chem. Eng. 3, 2750–2759. doi: 10.1016/j.jece.2015.05.010

Parshetti, G. K., Chowdhury, S., and Balasubramanian, R. (2015). Biomass derived low-cost microporous adsorbents for efficient CO_2 Capture. Fuel 148, 246–254. doi: 10.1016/j.fuel.2015.01.032

Sangchoom, W., and Mokaya, R. (2015). Valorization of lignin waste: carbons from hydrothermal carbonization of renewable lignin as superior sorbents for CO_2 and hydrogen storage. ACS Sustain. Chem. Eng. 3, 1658–1667. doi: 10.1021/acssuschemeng.5b00351

Serafin, J., Baca, M., Biegun, M., Mijowska, E., Kaleńczuk, R. J., and Sreńscek-Nazzal, J., et al. (2019). Direct conversion of biomass to nanoporous activated biocarbons for high CO_2 adsorption and supercapacitor applications, Appl. Surf. Sci. 497, 143722. doi: 10.1016/j.apsusc.2019.143722

Sevilla, M., and Fuertes, A. B. (2011). Sustainable porous carbons with a superior performance for CO_2 capture, Energy Environ. Sci. 4, 1765–1771. doi: 10.1039/C0EE00784F

Silvestre-Albero, A., Silvestre-Albero, J., Martínez-Escandell, M., and Rodríguez-Reinoso, F. (2014) Micro/mesoporous activated carbons derived from polyaniline: promising candidates for CO_2 adsorption. Ind. Eng. Chem. Res. 53, 15398−15405. doi: 10.1021/ie5013129

Vargas, D. P., Giraldo, L., Silvestre-Albero, J., and Moreno-Piraján, J. C. (2011). CO_2 adsorption on binderless activated carbon monoliths. Adsorption 17, 497–504. doi: 10.1007/s10450-010-9309-z

Wahby, A., Ramos-Fernández, J. M., Martínez-Escandell, M., Sepúlveda-Escribano, A., Silvestre-Albero, J. and Rodríguez-Reinoso, F. (2010). High-surface-area carbon molecular sieves for selective CO_2 adsorption. ChemSusChem. 3, 974–981. doi: 10.1002/cssc.201000083

Wang, J., Senkovska, I., Oschatz, M., Lohe, M. R., Borchardt, L., Heerwig, A., et al. (2013). Highly porous nitrogen-doped polyimine-based carbons with adjustable microstructures for CO_2 capture. J. Mater. Chem. A 1, 10951–10961. doi: 10.1039/c3ta11995e

Wei, H., Deng, S., Hu, B., Chen, Z., Wang, B., Huang, J., et al. (2012). Granular bamboo-derived activated carbon for high CO_2 adsorption: The Dominant Role of Narrow Micropores. ChemSusChem. 5, 2354–2360. doi: 10.1002/cssc.201200570
